# Comparison of acupuncture intervention from the acute phase or the non-acute phase in patients with peripheral facial paralysis: a systematic review and meta-analysis

**DOI:** 10.3389/fneur.2025.1690231

**Published:** 2025-11-25

**Authors:** Lele Zhang, Min Ye, Hongyu Xie, Yingju Hu, Aihong Yuan

**Affiliations:** Department of Acupuncture, The First Affiliated Hospital of Anhui University of Chinese Medicine, Hefei, Anhui, China

**Keywords:** acupuncture, timing, peripheral facial paralysis, systematic review, meta-analysis

## Abstract

**Background:**

The optimal timing of acupuncture intervention in peripheral facial paralysis (PFP) remains uncertain. This study compared the effectiveness of acupuncture administered during the acute versus non-acute phases of PFP.

**Methods:**

A systematic search of eight databases was conducted for relevant literature published from inception until July 1, 2025. This study included randomized controlled trials (RCTs) that met the predetermined inclusion criteria. The evaluated outcomes encompassed the clinical effective rate, House-Brackmann Facial Nerve Grading Scale (H-B scale), Facial Disability Index (FDI), cure time, Portmann Simple Score Scale (Portmann score), and non-cure rate at 1-month follow-up. Study selection and data extraction were performed independently by two reviewers. This study utilized the Cochrane Risk of Bias tool and the GRADE framework to assess methodological quality and evidence certainty, respectively. Data analysis was conducted using Review Manager 5.4 and Stata 15.0, with results expressed as relative risk (RR) or mean difference (MD) with corresponding 95% confidence intervals (95% CI).

**Results:**

This meta-analysis included 15 randomized controlled trials involving 771 patients, with the majority demonstrating an unclear or low risk of bias. Pooled results indicated that acupuncture as an adjunctive therapy significantly improved the clinical effective rate (RR = 1.11, 95% CI [1.06, 1.16], *p* < 0.0001), corresponding to an 11% relative increase in the probability of treatment success. Significant improvements were also observed in facial nerve function, as measured by the H-B scale (MD = −0.56, 95% CI [−0.92, −0.20], *Z* = 3.03, *p* = 0.002), FDI Physical Function subscale (MD = 2.57, 95% CI [0.54, 4.59], *Z* = 2.48, *p* = 0.01) and Portmann score (MD = 3.69, 95% CI [0.50, 6.87], *Z* = 2.27, *p* = 0.02). Additionally, acupuncture substantially reduced cure time (MD = −10.71 days, 95% CI [−16.33, −5.09], *p* = 0.0002). In contrast, neither the FDI Social Function subscale (MD = −0.89, 95% CI [−2.48, 0.71], *p* = 0.28) nor the non-cure rate at 1-month follow-up (RR = 0.63, 95% CI [0.21, 1.91], *p* = 0.42) showed statistically significant improvement. The certainty of evidence was rated as low for most outcomes according to GRADE criteria.

**Conclusion:**

This synthesis provides preliminary evidence that integrating acupuncture with Western medicine during the acute phase of PFP may enhance clinical response rates and accelerate facial functional recovery. However, these conclusions are tempered by methodological limitations observed in the included trials, particularly concerning potential biases and small sample sizes. Consequently, these findings highlight the imperative for more rigorously designed, large-scale randomized controlled trials to establish definitive evidence for clinical application.

**Systematic review registration:**

https://www.crd.york.ac.uk/PROSPERO/myprospero, Unique Identifier: CRD420251084963.

## 1 Introduction

Peripheral facial paralysis (PFP) represents the most common disorder of the facial nerve, with a reported incidence ranging from 11.5 to 53.3 per 100,000 individuals (1). The condition typically presents with impaired or lost voluntary movement and expressive function in the unilateral facial muscles. Standard treatment typically consists of oral corticosteroids and neurotrophic medications, with evidence supporting early initiation to improve recovery rates (2, 3). Although approximately 60% of patients achieve complete recovery within 2 weeks to 3 months of treatment, nearly 30% continue to experience varying degrees of sequelae despite timely and comprehensive intervention (4). This persistent therapeutic challenge has generated considerable clinical interest in developing additional interventions that can enhance recovery from PFP.

Acupuncture, a traditional Chinese therapy, represents a beneficial therapeutic approach for PFP. Evidence indicates that acupuncture intervention can alleviate facial symptoms, enhance cure rates, and reduce recovery time in PFP patients (5). Furthermore, it demonstrates favorable effects in managing PFP sequelae (6). The World Health Organization has recognized PFP as an indication for acupuncture therapy, and previous systematic reviews and meta-analyses have provided preliminary confirmation of its efficacy (7). These therapeutic effects are supported by proposed biological mechanisms, including improved local microcirculation to ameliorate neuro-edema, modulation of inflammatory responses, and facilitation of functional reorganization within the central nervous system to restore facial motor control (8–10).

The optimal timing for acupuncture intervention in PFP remains a subject of ongoing debate. Conventional medical guidelines often recommend initiating acupuncture during the recovery phase, typically recommending initiation approximately 1 week following symptom onset, based on safety considerations (3, 11). Conversely, emerging evidence supports the suitability of early acupuncture intervention, with patients receiving treatment within 7 days of onset demonstrating lower recurrence rates, shorter recovery times, higher cure rates, and improved overall prognoses (12, 13). This clear divergence in clinical recommendations underscores the necessity for comprehensive evidence synthesis to evaluate the potential benefits of early-phase acupuncture intervention for PFP patients.

Previous meta-analyses addressing this clinical question present several methodological limitations. Certain studies incorporated patients across all disease phases or exclusively during non-acute stages, thereby precluding precise assessment of phase-specific treatment effects (7, 14). Other investigations examining acupuncture and moxibustion during the acute phase were published earlier and exhibited substantial heterogeneity in therapeutic protocols (15, 16). Furthermore, a previously published meta-analysis focusing specifically on the timing of acupuncture intervention for PFP was subsequently retracted (17). These identified gaps in the current evidence base necessitate an updated and methodologically rigorous systematic review. The present study was therefore designed to systematically evaluate and compare the effectiveness of acupuncture intervention in PFP patients from the acute or non-acute phase, with the ultimate objective of providing robust evidence to inform clinical decision-making regarding optimal intervention timing.

## 2 Methods

### 2.1 Protocol and registration

This systematic review was conducted in accordance with the Preferred Reporting Items for Systematic Reviews and Meta-Analyses (PRISMA) 2020 guidelines (18). The study protocol was prospectively registered in the International Prospective Register of Systematic Reviews (PROSPERO) under registration number CRD420251084963.

### 2.2 Search strategy

The literature search encompassed eight electronic databases from their inception through July 1, 2025. The selected databases comprised four international sources (PubMed, Embase, Cochrane Library, and Web of Science) and four Chinese databases (China National Knowledge Infrastructure, China BioMedical Literature Database, Wanfang Digital Journals, and VIP database for Chinese Technical Periodicals). The search imposed no restrictions on publication period, ethnicity, or language. Four review authors (LZ, MY, HX, YH) collaboratively developed and executed the comprehensive search strategy. Supplementary Table S1 provides the complete search terms utilized in this study.

### 2.3 Inclusion criteria

Participants: The review included studies involving patients diagnosed with acute idiopathic peripheral facial paralysis, with the acute phase defined as symptom onset within 7 days. No restrictions were applied regarding age, ethnicity, geographic location, or educational background. Studies involving patients with secondary causes of facial paralysis were excluded, including but not limited to infectious etiologies (e.g., Ramsay Hunt syndrome, Lyme disease, mumps, otitis media) and traumatic injuries (e.g., temporal bone fracture, post-surgical damage).

Interventions: During the acute phase, the experimental group received acupuncture, which was administered in combination with Western medicine and complementary therapies. The latter included rehabilitation exercises, microwave therapy, ultrashort wave therapy, and infrared radiation. The specific complementary therapies employed, along with their mode of administration (symmetrical or asymmetrical between groups), were recorded as part of the intervention profile for each primary study (Table 1). While this clinical heterogeneity represents a study limitation, the primary objective of this review was to evaluate the timing of acupuncture initiation within the context of these real-world treatment regimens. Following the acute phase, acupuncture therapy was continued as a subsequent treatment, either as a standalone intervention or in combination with other therapies. The duration and specific regimen of all acupuncture interventions were documented.

**Table 1:** The basic characteristics of the included studies.

Study ID	Year	Sample size (E/C)	Mean Age (years) (E/C)	Course (days) (mean ± SD)		Experimental intervention		Control intervention		Intervention time	Outcome	Country
				E	C	Acute phase	Non-acute phase	Acute phase	Non-acute phase			
Xiao et al. (28)	2023	40/40	42.31/41.82	3.56 ± 1.39	3.71 ± 1.41	A + WM	A + WM	WM	A + WM	21 days	①②③	China
Ying et al. (30)	2020	80/80	42.27/41.56	2.38 ± 1.32	2.41 ± 1.15	A + WM	A + WM	WM	A + WM	27 days	①	China
Jinghua et al. (21)	2019	30/30	37/40	3.6 ± 1.4	4.1 ± 1.5	A + WM	A + EA + WM	WM	A + EA + WM	28 days	①②③④	China
Ying et al. (31)	2019	20/20	41.85/42.03	2.30 ± 0.85	2.25 ± 0.83	A + WM	A + WM + CT	WM	A + WM + CT	28 days	①②	China
Yun and Xiaoyang (33)	2019	30/30	34/34	2.1 ± 0.8	2.0 ± 0.8	A + CT	A + CT	WM	A + CT	21 days	①④⑤	China
Shuang et al. (26)	2018	30/30	33.53/34.70	≤3		A + WM	A + WM	WM	A + WM	28 days	①⑤	China
Ping and Yunxia (25)	2018	30/30	46.7/44.17	≤7		A + WM + CT	A + EA + WM	WM	A + EA + WM	56 days	①④	China
Simin (27)	2017	44/44	43.2/43.2	1.4 ± 0.3		A + WM + CT	A + WM	WM + CT	A + WM	30 days	①③	China
Bo et al. (19)	2016	32/30	41/42	2.06 ± 0.14	2.10 ± 0.76	A + WM	A + WM	WM	A + WM	28 days	①②⑤	China
Yanwu and Wei (29)	2013	39/36	43.15/41.56	≤2		A + WM + CT	A + WM + CT	WM + CT	A + WM + CT	28 days	①⑥	China
Dongyun and Haiyun (20)	2013	30/30	35/36	1.3 ± 0.5	1.5 ± 0.4	A + WM + CT	A + WM + CT	WM	A + WM + CT	24 days	①	China
Nianwen (24)	2012	42/38	51.43/50.15	1		A + WM	A + WM	WM	A + WM	14 days	①	China
Lifang et al. (23)	2011	86/84	45.32/46.42	2.05 ± 1.01	2.06 ± 1.34	A + WM + CT	A + WM + CT	WM + CT	A + WM + CT	42 days	①②④	China
Yufeng et al. (32)	2011	40/40	42.3/43.1	≤7		A + WM	A + WM	WM	A + WM	21 days	①	China
Lian et al. (22)	2010	44/42	37.85/37.53	1.43 ± 1.02	1.43 ± 0.74	A + CT	A + CT	WM	A + CT	28 days	①⑥	China

E, experimental group; C, control group; A, acupuncture; WM, Western Medicine; EA, electroacupuncture; CT, complementary therapy; ①: clinical effective rate; ②: House-Brackmann Facial Nerve Grading Scale; ③: Facial Disability Index; ④: cure time; ⑤: Portmann Simple Score Scale; ⑥: non-cure rate at 1-month follow-up.

Comparisons: During the acute phase, the control group received conventional treatment excluding acupuncture interventions. In the non-acute phase, both control and experimental groups received identical treatment regimens.

Outcome measures: The clinical effective rate served as the primary outcome. Secondary outcomes included the House-Brackmann Facial Nerve Grading Scale (H-B scale), Facial Disability Index (FDI), Portmann Simple Score Scale (Portmann score), non-cure rate at 1-month follow-up, and cure time.

Study design: The included studies were limited to randomized controlled trials (RCTs).

### 2.4 Exclusion criteria

The following were excluded: duplicate publications; studies with unavailable critical data or insufficient reporting of key outcomes; and secondary literature including reviews, dissertations, and conference proceedings.

### 2.5 Selection of studies and data extraction

Duplicate records were identified and removed using EndNote 21 software, based on title, author, and publication year. Two reviewers (MY and LZ) independently screened the titles and abstracts of all retrieved records. After obtaining full-text articles, the same reviewers independently assessed their eligibility for inclusion. The final study selection was determined through consensus between both reviewers. Any disagreements during the selection process were resolved through consultation with two additional reviewers (HX and YH).

Data extraction was performed independently by two reviewers (LZ and MY) using a standardized form. The extracted data were then cross-checked for accuracy. Discrepancies in data extraction were resolved through discussion, with unresolved issues adjudicated by the additional reviewers (HX and YH). The following data were extracted from each included study: first author, publication year, country, sample size, participant age and sex, detailed intervention and control protocols, treatment duration, outcome measures, and other relevant study characteristics.

### 2.6 Risk of bias and evidence quality assessment

Methodological quality of the included studies was assessed using the Cochrane Risk of Bias (RoB) tool, which evaluates seven specific domains and classifies overall risk as low, high, or some concerns. The quality of evidence for each outcome was evaluated using the GRADE (Grading of Recommendations, Assessment, Development, and Evaluations) framework. This process included creating evidence profile tables and categorizing evidence quality as high, moderate, low, or very low based on established criteria. Any disagreements in assessments were resolved through consensus discussions or third-party adjudication when necessary.

### 2.7 Statistical analysis

The statistical software Stata 15.0 and RevMan 5.4 were used to conduct the meta-analysis. For continuous variables, the mean difference was applied, and relative risk was used for dichotomous variables. The confidence interval (CI) for all effect sizes was set at 95%. A fixed-effects model was used when heterogeneity was not significant (*I^2^* ≤ 50% and *P* > 0.1). Conversely, a random-effects model was employed when significant heterogeneity was observed (*I^2^* > 50% and *p* < 0.1). Subgroup analyses were used to examine the possible impact of key variables on the outcomes. The robustness of the meta-analysis results was assessed using a sensitivity analysis. Quantitative and qualitative assessments of publication bias were performed using Egger’s test and visual inspection of funnel plot asymmetry. If publication bias was suspected, the trim-and-fill method was used to confirm whether publication bias had a significant effect on the results.

## 3 Results

### 3.1 Study selection

**Figure 1:**
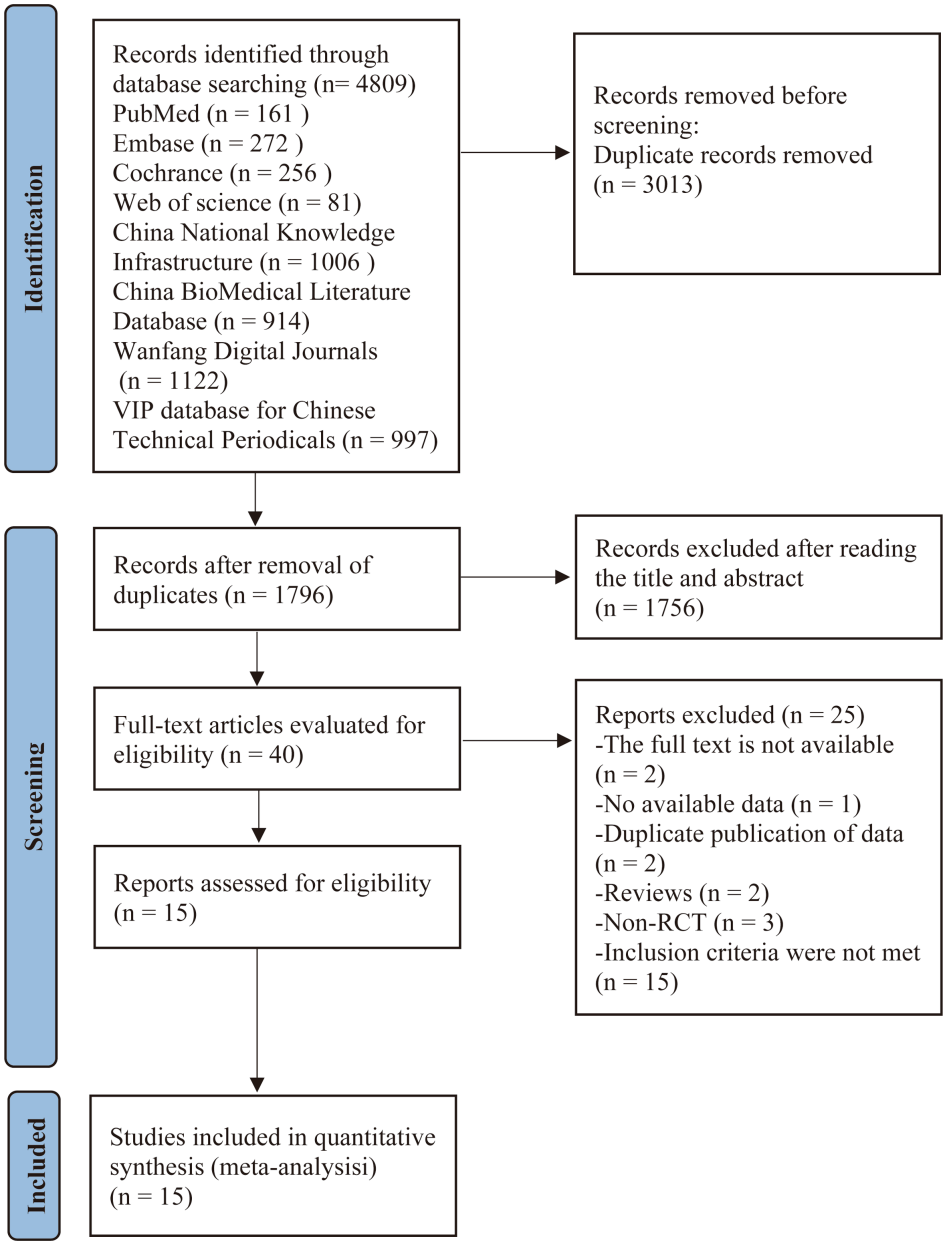
Flow diagram of study screening.

The initial literature search yielded 4,809 records. After removing 3,013 duplicates, 1,796 articles underwent title and abstract screening, which led to the exclusion of 1,756 records that did not meet the inclusion criteria. The remaining 40 articles underwent full-text review for eligibility assessment. Of these, 25 studies were excluded with reasons documented in Figure 1. Ultimately, 15 studies (19–33) qualified for inclusion in the systematic review and meta-analysis. The study selection process is detailed in the PRISMA flow diagram (Figure 1).

### 3.2 The basic characteristics of the included studies

Fifteen studies were included in this review (19–33), with sample sizes ranging from 40 to 170 participants (total 771 patients). All studies were conducted in China and published in Chinese between 2011 and 2023. Baseline characteristics showed no statistically significant differences between groups in any included study. Table 1 summarizes the key characteristics of the included studies.

### 3.3 The risk of bias assessment of included studies

**Figure 2:**
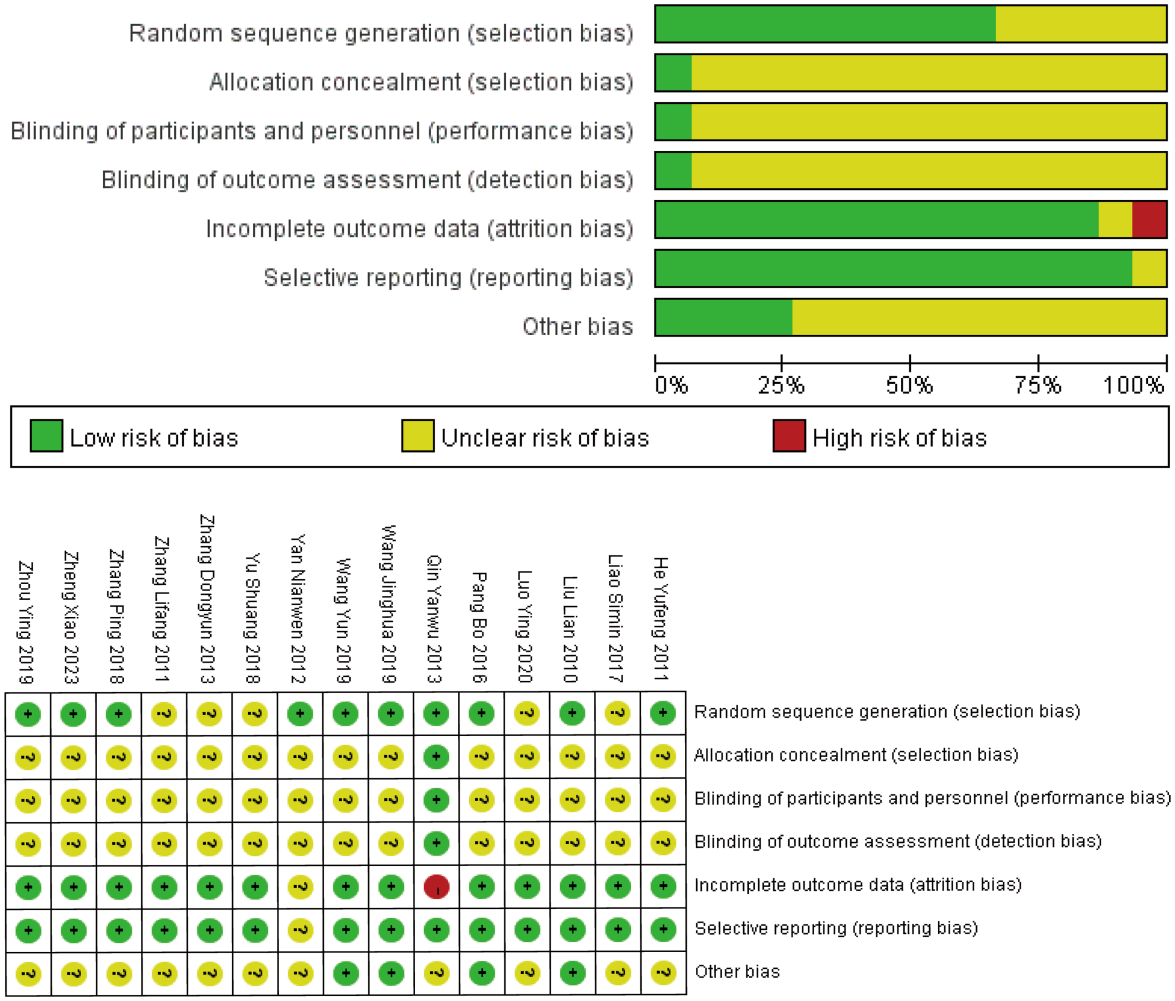
The risk of bias assessment for the included studies.

All 15 RCTs reported using randomization methods, although five studies (20, 23, 26, 27, 30) failed to specify the specific randomization techniques employed. Only one study (29) described adequate allocation concealment using the envelope method, while the remaining studies provided insufficient information on allocation concealment procedures. Blinding of participants, personnel, and outcome assessors was explicitly reported in only one study (29). One study (24) exclusively reported differences in outcome measures before and after treatment without other methodological details. Another study (29) did not account for drop-out cases in the analysis. Eleven studies were rated as having an unclear risk of bias in the “other bias” category due to insufficient reporting. The overall assessment indicated significant methodological concerns, primarily stemming from inadequate allocation concealment and lack of blinding procedures. The complete risk of bias assessment is presented in Figure 2.

### 3.4 Meta-analysis

The included studies were stratified into two subgroups according to whether acupuncture was combined with Western medicine during the acute phase: (1) acupuncture combined with Western medicine in the acute phase (19–21, 23–32); (2) acupuncture not combined with Western medicine in the acute phase (22, 33).

#### 3.4.1 Clinical effective rate

**Figure 3:**
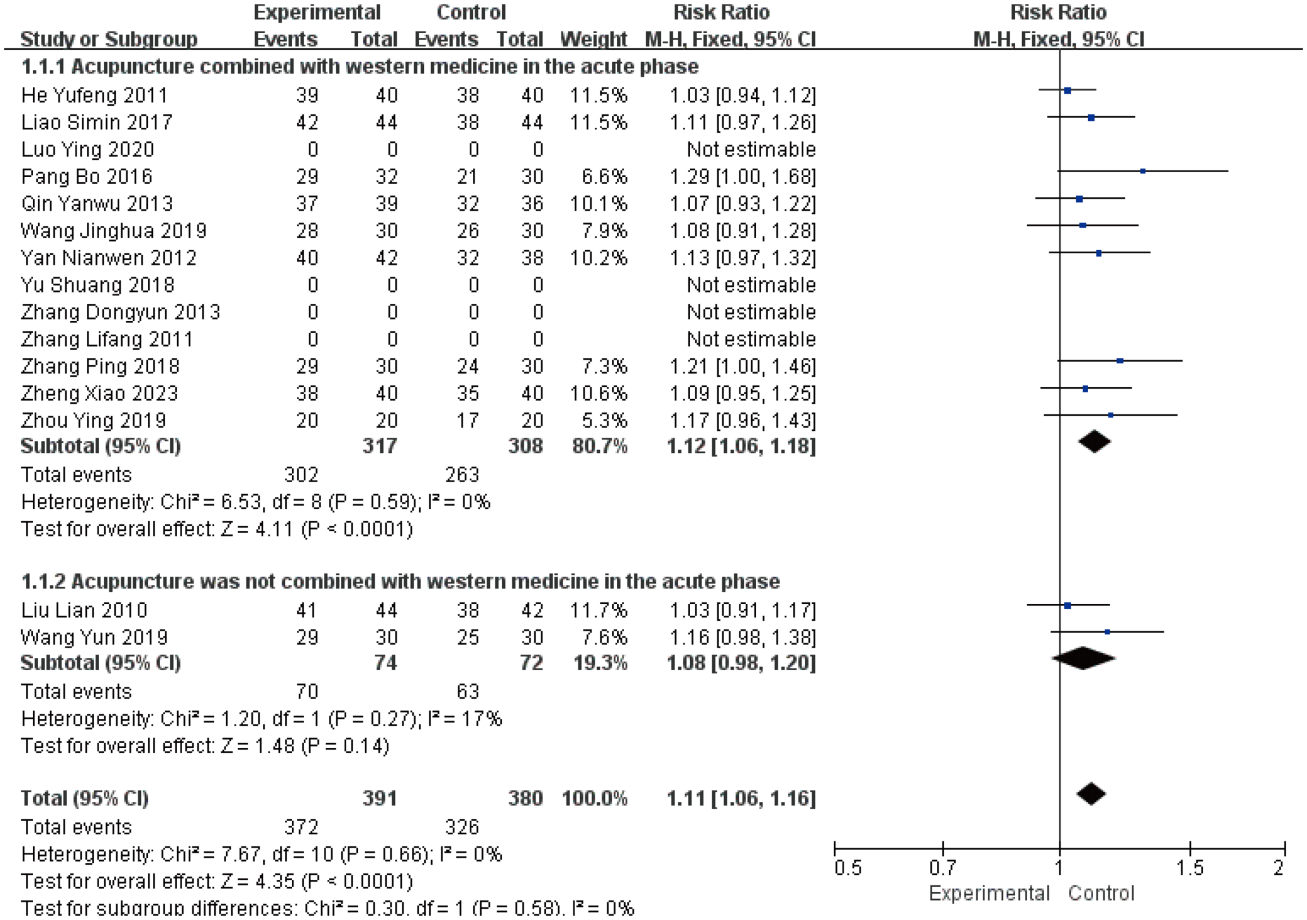
Forest plot for clinical effective rate.

**Figure 4:**
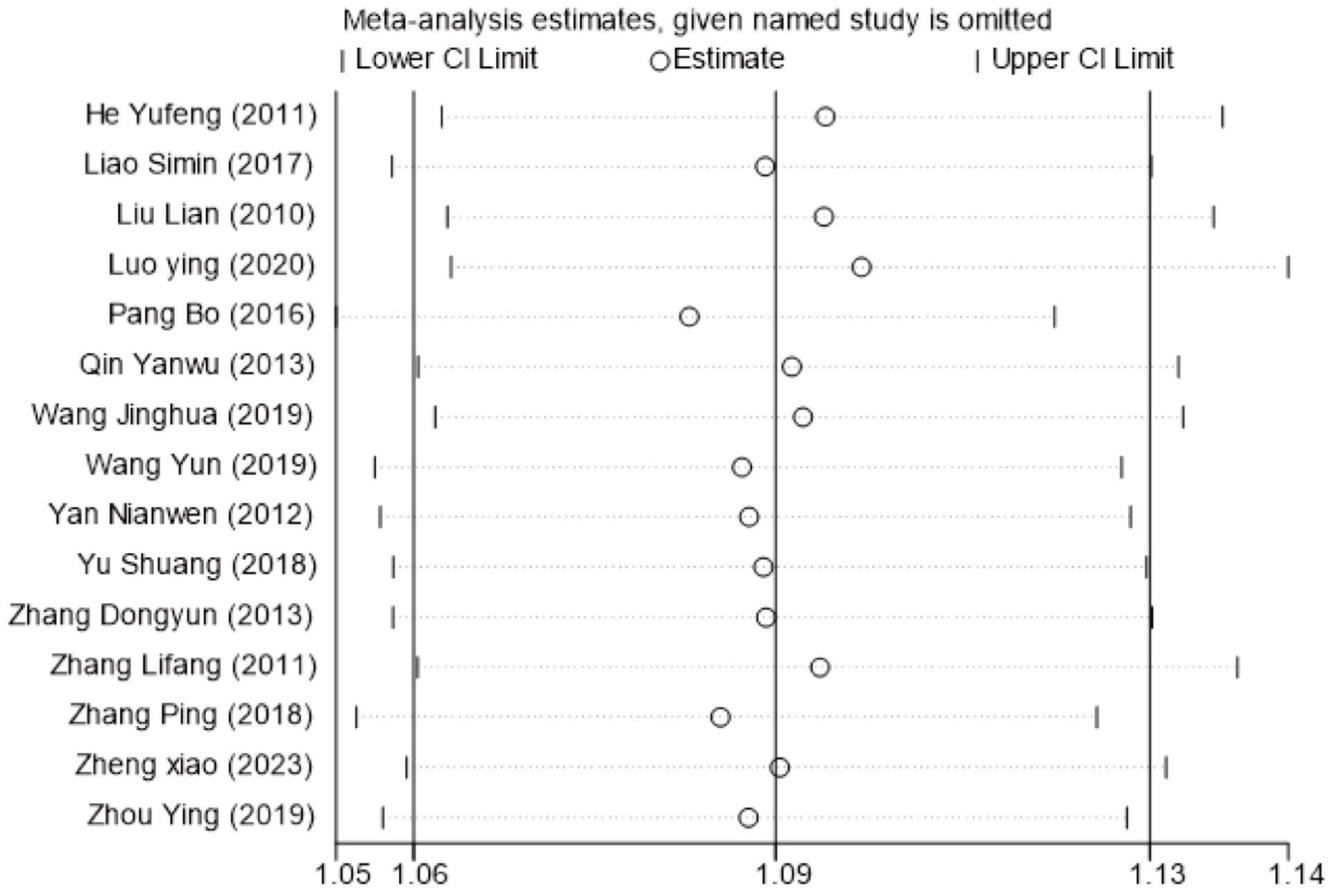
Sensitivity analysis for clinical effective rate.

The clinical effective rate was analyzed across all 15 included studies (19–33), encompassing 771 patients. Statistical analysis revealed no significant heterogeneity (*I*^2^ = 0%, *p* > 0.05), supporting the use of a fixed-effects model. The subgroup analysis results showed a Risk Ratio (RR) of 1.12 (*I^2^* = 0, 95%CI [1.06, 1.18], *P* < 0.0001) and another RR of 1.08 (*I^2^* = 17, 95%CI [0.98, 1.20], *p* = 0.14) (Figure 3). This suggests that while the combination therapy is clearly beneficial, the evidence is insufficient to conclude that acupuncture as a standalone treatment significantly outperforms Western medicine in terms of clinical effective rate. Sensitivity analysis confirmed the robustness of these results (Figure 4). Egger’s test suggested potential publication bias (*p* = 0.001); however, application of the trim-and-fill method confirmed that the pooled effect size remained robust (Supplementary Figures S1–S5).

#### 3.4.2 H-B scale scores

Five studies (19, 21, 23, 28, 31) involving 412 patients assessed facial nerve function using the H-B scale. Analysis revealed substantial heterogeneity (*I*^2^ = 65%, *p* = 0.02), prompting further investigation. Sensitivity analysis demonstrated that sequential exclusion of individual studies neither altered the direction of the pooled effect size nor produced a substantial or consistent reduction in heterogeneity. Consequently, no single study was identified as a major contributor to the observed heterogeneity through this method (Supplementary Figures S6, S7).

**Figure 5:**
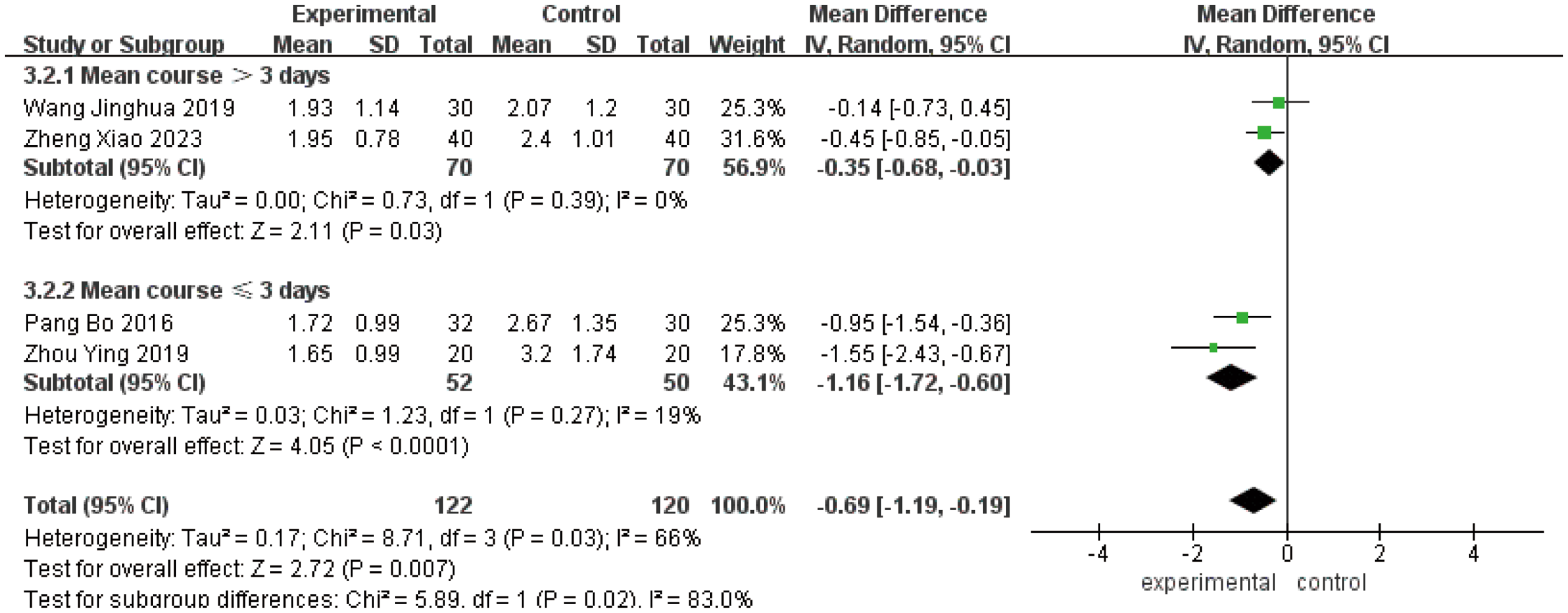
Forest plot for H-B scale scores.

**Figure 6:**
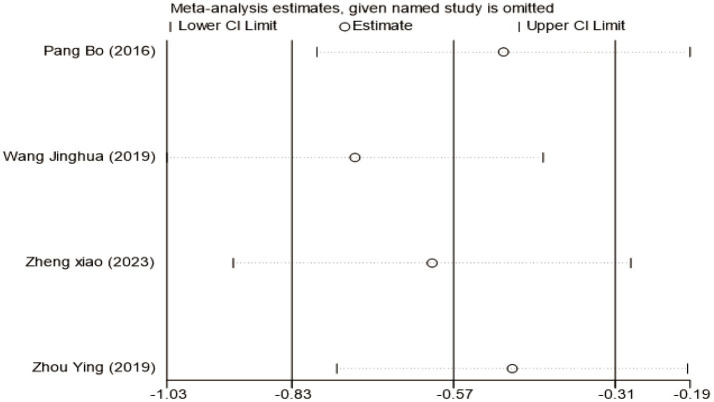
Sensitivity analysis for H-B scale scores.

To investigate the sources of heterogeneity in H-B scale outcomes, a subgroup analysis was conducted based on mean disease course (≤3 days vs. >3 days). While this approach partially explained the observed heterogeneity, subsequent sensitivity analysis identified the study by Lifang et al. (23) as a major contributor (Supplementary Figure S8). This study employed a substantially longer intervention time (42 days) compared to others in the same subgroup (typically 21–28 days). Exclusion of this study reduced heterogeneity within subgroups from *I*^2^ = 81% to *I*^2^ = 19%, confirming intervention time as a key source of heterogeneity (Figure 5). The pooled analysis of the remaining studies demonstrated a significant mean difference of −0.69 (95% CI [−1.19, −0.19], *p* < 0.05), indicating that acupuncture combined with Western medicine during the acute phase provides superior improvement in H-B scale scores compared to Western medicine alone. Sensitivity analysis confirmed the stability of these findings (Figure 6), and Egger’s test showed no significant publication bias (*p* = 0.374; Supplementary Figures S9–S11).

#### 3.4.3 FDI scores

Three studies involving 228 patients evaluated outcomes using the Facial Disability Index (21, 27, 28), which comprises two subscales: the Physical Function subscale (FDIP) and Social Function subscale (FDIS).

**Figure 7:**
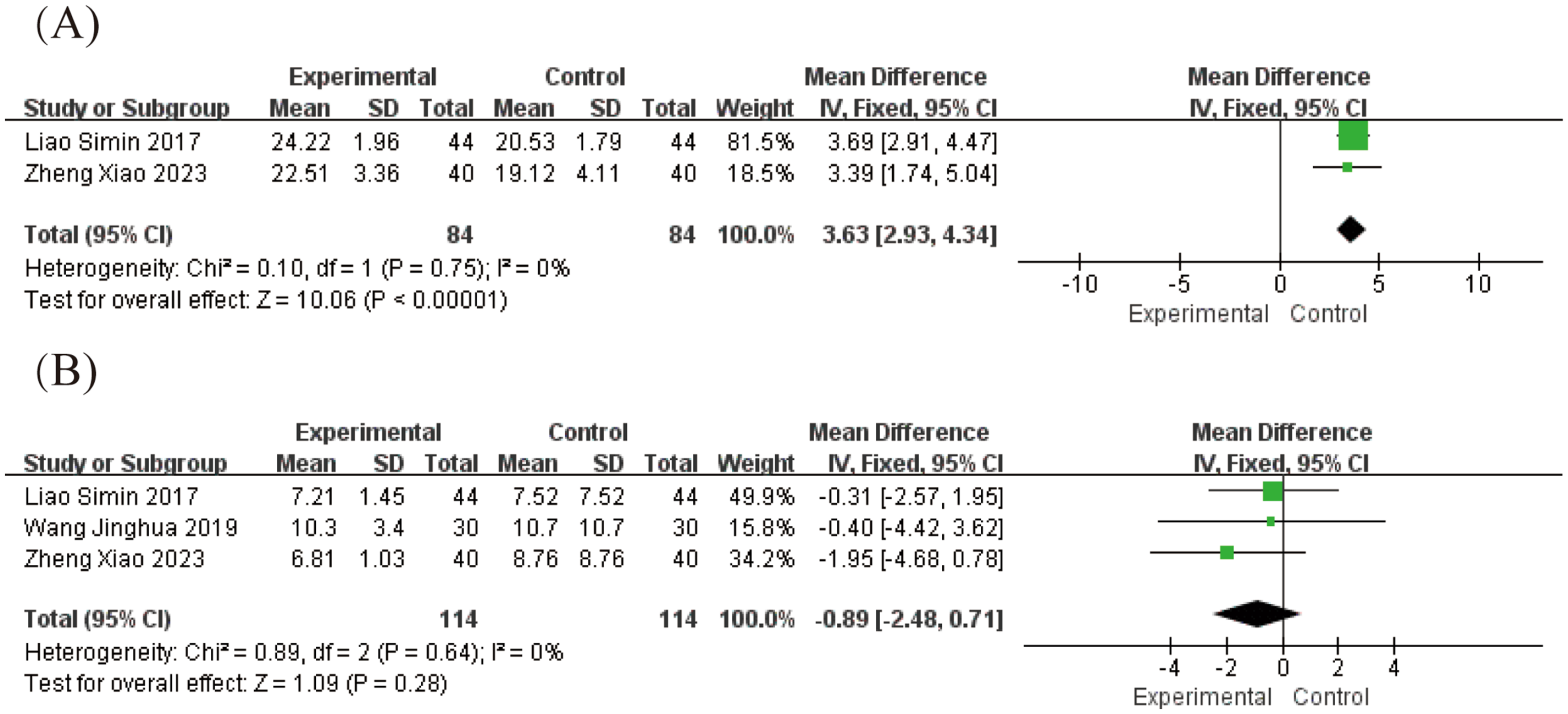
Forest plot for FDI scores. **(A)** Forest plot for FDIP scores, **(B)** Forest plot for FDIS scores.

For FDIP scores, meta-analysis revealed substantial heterogeneity (*I*^2^ = 89%, *p* = 0.0002). Sensitivity analysis identified the study by Wang Jinghua (21) as the primary source of heterogeneity, which uniquely incorporated electroacupuncture unlike other studies in this subgroup (Supplementary Figures S12, S13). Exclusion of this study reduced heterogeneity to *I*^2^ = 0%, confirming acupuncture modality as a key factor contributing to heterogeneity (Figure 7A). The pooled analysis demonstrated a significant mean difference of 3.63 (95% CI [2.93, 4.34], *p* < 0.05), indicating that acute-phase acupuncture combined with Western medicine provides superior improvement in physical function compared to Western medicine alone. Funnel plot inspection showed no evident publication bias (Supplementary Figure S14).

For FDIS scores, analysis of social function outcomes showed no significant heterogeneity (*I*^2^ = 0%, *p* = 0.64), supporting the use of a fixed-effects model. The results showed a non-significant mean difference of −0.89 (95% CI [−2.48, 0.71], *p* = 0.28), indicating that the addition of acupuncture to Western medicine did not provide measurable benefits for social function and well-being within the study timeframe (Figure 7B). Egger’s test confirmed the absence of significant publication bias (*p* = 0.771; Supplementary Figures S15–S17).

#### 3.4.4 Portmann score

Portmann score assessments were conducted at multiple time points. Two studies (19, 33) involving 122 patients reported scores at 7 and 14 days post-onset, while three studies (19, 26, 33) with 182 patients provided data at 28 days post-onset.

**Figure 8:**
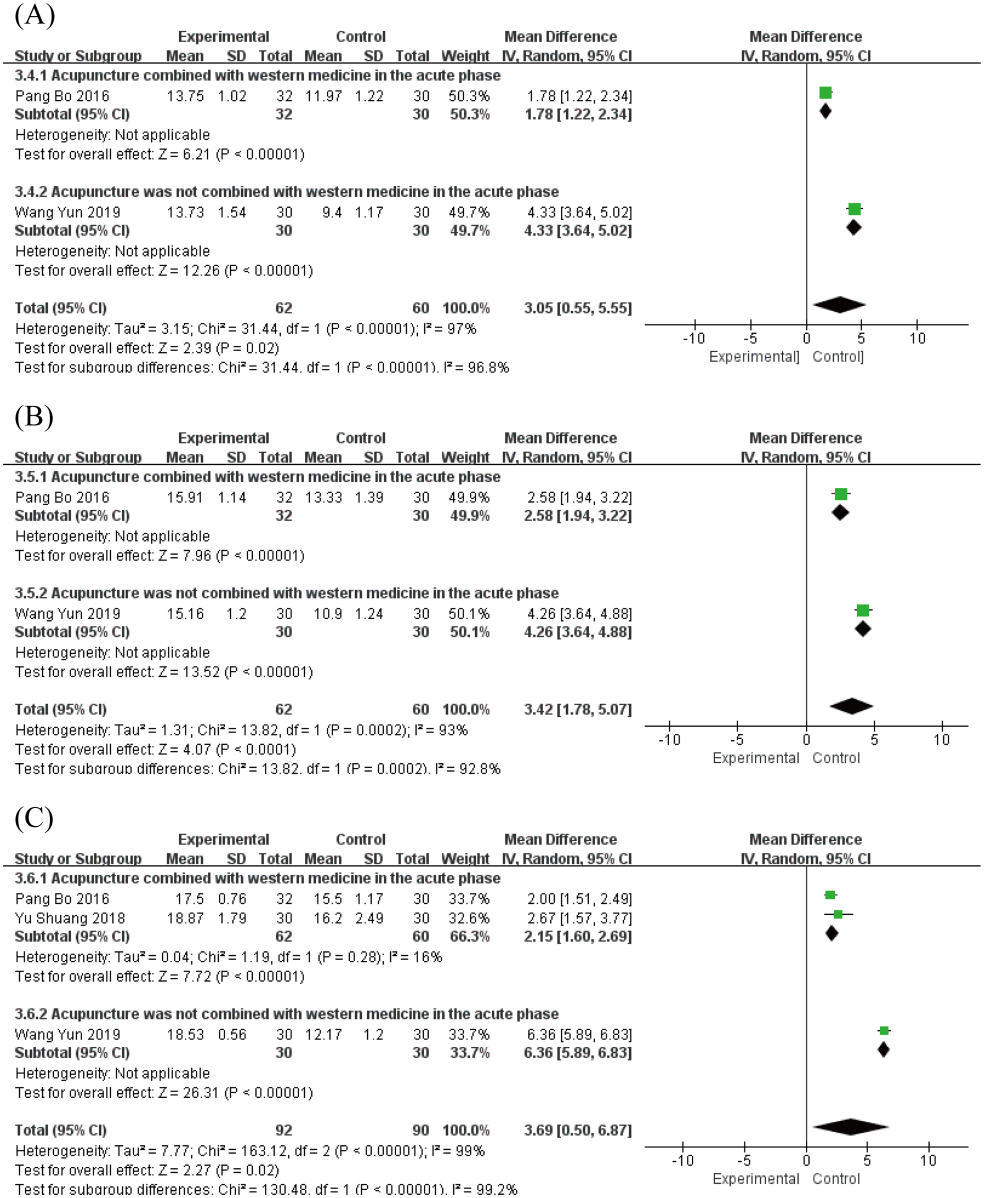
Forest plot for Portmann score. **(A)** Forest plot for 7 days post-onset. **(B)** Forest plot for 14 days post-onset. **(C)** Forest plot for 28 days post-onset.

At 7 days post-onset, analysis revealed substantial heterogeneity (*I*^2^ = 97%, *p* < 0.00001) using a random-effects model, showing a significant mean difference of 3.05 (95% CI [0.55, 5.55], *p* = 0.02; Figure 8A). By 14 days post-onset, heterogeneity remained high (*I*^2^ = 93%, *p* = 0.0002) with a mean difference of 3.42 (95% CI [1.78, 5.07], *p* < 0.0001; Figure 8B). At 28 days post-onset, significant heterogeneity persisted (*I*^2^ = 99%, *p* < 0.00001) with a mean difference of 3.69 (95% CI [0.50, 6.87], *p* = 0.02; Figure 8C). The pre-specified subgroup analysis, based on whether acupuncture was combined with Western medicine during the acute phase, revealed a statistically significant subgroup difference (Figures 8A–C). The significant between-subgroup difference strongly suggests that the therapeutic strategy (acupuncture alone vs. acupuncture combined with Western medicine) is a major source of the observed statistical heterogeneity, explaining the variability in treatment effects across studies. These findings consistently demonstrate the superior efficacy of acute-phase acupuncture intervention in improving Portmann scores from the first to fourth week compared to Western medicine alone. Sensitivity analysis confirmed result robustness, and Egger’s test (*p* = 0.073) indicated no significant publication bias (Supplementary Figures S18–S21).

#### 3.4.5 Cure time

**Figure 9:**
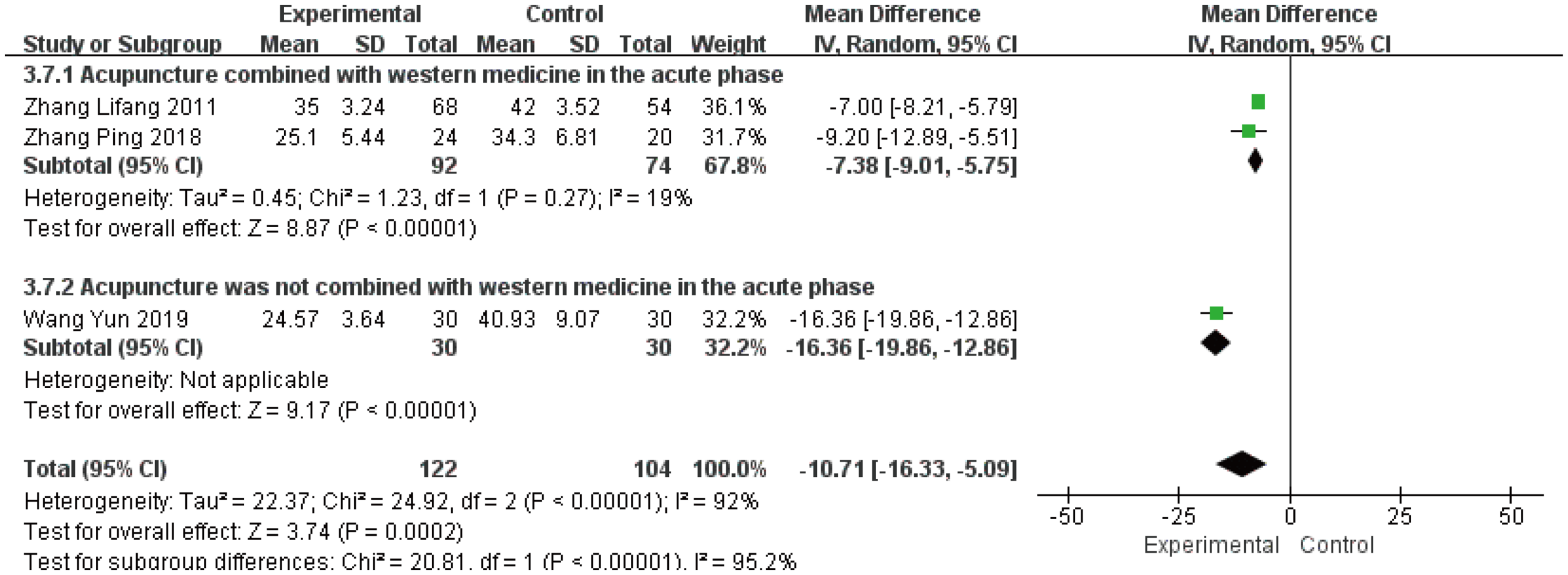
Forest plot for cure time.

Three studies (23, 25, 33) involving 226 patients evaluated cure time as a secondary outcome. The initial analysis demonstrated substantial heterogeneity (*I*^2^ = 92%, *p* < 0.00001), leading to the use of a random-effects model. The pooled results showed a significant mean difference of −10.71 days (95% CI [−16.33, −5.09], *p* = 0.0002), indicating that acute-phase acupuncture intervention substantially reduced cure time compared to Western medicine alone (Figure 9). Subgroup analysis based on combination with Western medicine during the acute phase effectively resolved the observed heterogeneity. The heterogeneity within each subgroup was substantially reduced (*I*^2^ = 19%), and the test for subgroup differences reached statistical significance (*P* for subgroup difference < 0.05). This demonstrates that the treatment strategy – whether acupuncture was integrated with Western medicine – represents a key factor explaining the heterogeneity in reported cure times. Sensitivity analysis confirmed the robustness of these findings, and Egger’s test (*p* = 0.836) indicated no significant publication bias (Supplementary Figures S22–S25).

#### 3.4.6 Non-cure rate at 1-month follow-up

**Figure 10:**
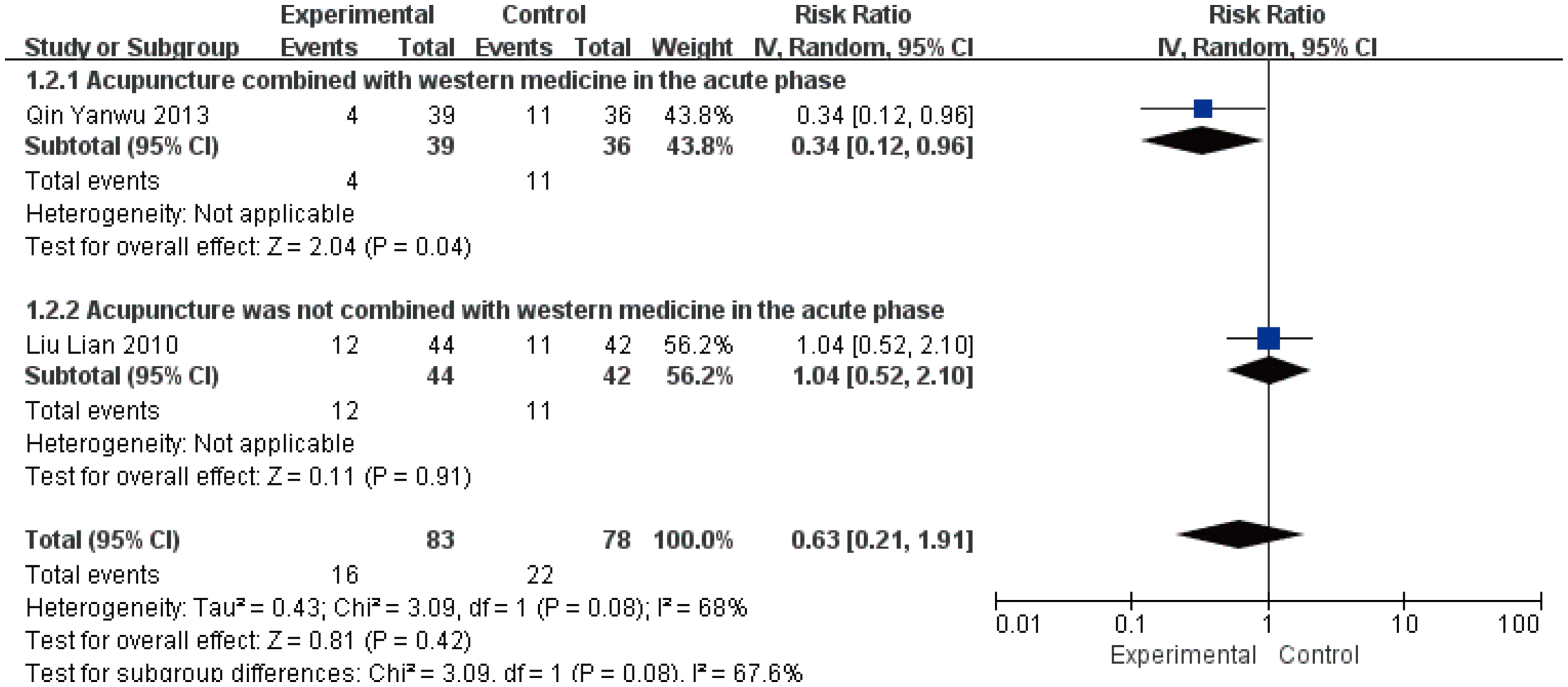
Forest plot for non-cure rate at 1-month follow-up.

Two studies involving 161 patients assessed the non-cure rate at 1-month follow-up (22, 29). Quantitative synthesis revealed moderate heterogeneity (*I*^2^ = 68%, *p* = 0.08), warranting the use of a random-effects model. Subgroup analysis demonstrated divergent treatment effects: acupuncture combined with Western medicine showed a significant reduction in non-cure rate (RR = 0.34, 95% CI [0.12, 0.96], *p* = 0.04), while acupuncture alone showed no significant effect (RR = 1.04, 95% CI [0.52, 2.10], *p* = 0.91) (Figure 10). Although the test for subgroup differences did not reach conventional statistical significance (*p* = 0.08), the contrasting point estimates between subgroups (risk reduction versus no effect) suggest that combination with Western medicine may be an important effect modifier for long-term outcomes. The wide confidence intervals crossing the line of no effect, particularly in the context of the limited number of available studies, indicate that the current evidence remains too uncertain to draw definitive conclusions regarding acupuncture’s long-term preventive effect on treatment failure. The non-significant subgroup difference likely reflects insufficient statistical power rather than the absence of a true differential treatment effect.

### 3.5 Grade of evidence quality

The GRADE tool was used to assess the quality of the evidence. The assessment results showed that the evidence quality was moderate or low (Table 2). Further details are provided in Supplementary Table S2.

**Table 2:** GRADE evidence profile in the meta-analysis.

Outcomes	Grade
Clinical effective rate	Moderate
House-Brackmann Facial Nerve Grading Scale	Moderate
Facial Disability Index-Physical	Low
Facial Disability Index-Social	moderate
Portmann Simple Score Scale on 7 days after onset	Low
Portmann Simple Score Scale on 14 days after onset	Low
Portmann Simple Score Scale on 28 days after onset	Low
Cure time	Low
Non-cure rate at 1-month follow-up	Very low

## 4 Discussion

This meta-analysis evaluated the efficacy of acupuncture initiated during acute versus non-acute phases of peripheral facial paralysis (PFP) to address existing clinical controversies. The synthesized evidence demonstrates that acupuncture combined with Western medicine during the acute phase yields significantly better outcomes than Western medicine alone across multiple domains, including facial nerve function recovery, cure time acceleration, and certain long-term efficacy measures. In contrast, acupuncture monotherapy did not demonstrate statistically significant advantages over conventional treatment for most outcomes. Furthermore, neither combination therapy nor acupuncture alone showed significant benefits in improving psychosocial function compared to control interventions.

Analysis of acupuncture monotherapy revealed a distinct pattern of outcomes. While acute-phase acupuncture alone demonstrated significant improvements in Portmann scores and reduction in cure time compared to Western medicine—findings consistent with previous research (7, 14). However, it failed to show statistically significant advantages in clinical effective rate, FDIS scores, or 1-month non-cure rates. These mixed results suggest that while acupuncture monotherapy may accelerate certain aspects of physiological recovery, its efficacy in improving broader quality-of-life dimensions and preventing long-term poor outcomes remains unestablished. Future investigation through adequately powered RCTs should prioritize both standardized acupuncture protocols and comprehensive outcome assessment to clarify these differential treatment effects.

Substantial statistical heterogeneity observed across several outcomes prompted comprehensive post-hoc analyses. For H-B scale scores, both disease course (≤3 days vs. >3 days) and intervention time emerged as significant contributors to heterogeneity. The critical neuroprotective window during early (within the first 3 days) nerve injury pathogenesis, characterized by dynamic inflammatory edema resolution, may explain the differential outcomes based on intervention timing (34). Similarly, extended treatment duration likely influences recovery trajectories through cumulative effects (35). FDIP score heterogeneity was primarily explained by electroacupuncture application during the non-acute phase. The continuous neurological stimulation provided by electroacupuncture may enhance nerve remodeling and functional recovery beyond manual acupuncture capabilities (36). Most consistently, treatment strategy—whether combining acupuncture with Western medicine—proved to be a fundamental effect modifier across multiple outcomes (clinical effective rate, Portmann score, cure time, and non-cure rate). This pattern suggests synergistic interaction, where acupuncture may improve local microcirculation and modulate neuro-inflammation to enhance drug delivery, while Western medicine establishes a foundational neuroprotective environment (37–39).

Despite these insights, residual heterogeneity within some subgroups persists, which may be attributed to other unmeasured factors such as subtle differences in acupuncture technique (e.g., needle manipulation, deqi sensation), variations in baseline severity of paralysis, and patient-specific characteristics. Nevertheless, the application of a random-effects model and the stability of results in sensitivity analyses support the robustness of our primary conclusions. Except for the clinical effective rate, no significant publication bias was detected for the other outcomes using funnel plots and Egger’s test. The trim-and-fill method was used to assess the impact of publication bias on the results, which showed statistical significance, indicating that the pooled effect size for the clinical effective rate was robust and not significantly affected by publication bias.

While a previous meta-analysis (40) indicated the potential superiority of combined acupuncture and Western medicine for PFP, its inclusion of patients with disease durations ranging from 1 to 14 days limits direct comparability with the current analysis, which specifically focused on the acute phase (≤7 days). Furthermore, this review differs from earlier meta-analyses (15, 16) that categorized diverse interventions—including acupuncture, acupuncture with moxibustion, and electroacupuncture—as equivalent acute-phase treatments, thereby introducing substantial clinical heterogeneity. In contrast, the current analysis employed more stringent inclusion criteria and maintained greater consistency in defining acupuncture interventions, representing a methodological refinement that enhances the validity of the findings.

Current evidence suggests that the therapeutic benefits of acupuncture for PFP may be mediated through multiple physiological pathways (41): the underlying mechanisms include enhanced local blood flow and microcirculatory perfusion that facilitate tissue repair, immunomodulatory effects that mitigate inflammatory responses, and promotion of cortical functional reorganization within the central nervous system to restore coordinated facial muscle control; when integrated with Western medicine, acupuncture may further improve drug delivery to affected tissues through its microcirculatory effects, while concurrently, Western pharmacotherapy may amplify the immunoregulatory benefits of acupuncture. Subgroup analyses from this review indicate that initiating combined acupuncture and Western medicine therapy during the acute phase represents a particularly promising strategy, demonstrating superior long-term outcomes and more consistent therapeutic effects compared to either intervention alone. These findings provide an evidence-based rationale for integrating acupuncture into conventional treatment protocols during the critical early stage of PFP, potentially offering a more comprehensive approach to managing this condition.

## 5 Limitations and prospects

Several limitations should be considered when interpreting these findings, which collectively account for the predominantly low or very low quality of evidence according to GRADE assessments. Methodological constraints in the included trials, particularly inadequate blinding procedures, unclear allocation concealment, and limited sample sizes, introduce potential biases and reduce precision in effect estimates. Furthermore, substantial clinical heterogeneity was observed due to variations in acupuncture implementation, including point selection, needle manipulation techniques, and adjunctive use of electroacupuncture, compounded by the absence of standardized treatment protocols. These factors complicate direct comparisons between studies and hinder identification of optimal therapeutic parameters. Generalizability of the results is constrained by the exclusive inclusion of studies conducted in China and published in Chinese, creating potential regional and language biases. Additionally, the scarcity of long-term follow-up data limits understanding of the sustained benefits of acute-phase acupuncture intervention.

Consequently, while the current evidence suggests that combining acupuncture with Western medicine during the acute phase represents a promising therapeutic approach, these findings should be considered preliminary and hypothesis-generating. To address these limitations and enhance evidence quality, future research should prioritize: (1) large, multi-center randomized trials with pre-specified sample size calculations; (2) rigorous methodological implementation including proper blinding and detailed randomization reporting; (3) development and adherence to standardized acupuncture protocols specifying core points, stimulation parameters, and treatment schedules; and (4) incorporation of objective outcome measures with mandatory long-term follow-up assessments to fully characterize treatment durability.

## 6 Conclusion

This study provides preliminary evidence that acupuncture combined with Western medicine during the acute phase may yield superior benefits for PFP recovery compared to Western medicine alone. Although evidence regarding acupuncture monotherapy remains inconclusive and these findings require validation through more rigorous investigation, these results support considering acupuncture as an adjunctive therapy within the conventional treatment window. The current findings provide a foundation for designing future definitive clinical trials to establish optimal treatment strategies for PFP management.

## Data Availability

The original contributions presented in the study are included in the article/Supplementary material, further inquiries can be directed to the corresponding author.
