# Supplementary material for: Comparison of acupuncture intervention from the acute phase or the non-acute phase in patients with peripheral facial paralysis: a systematic review and meta-analysis
Source: Front Neurol. 2025 Nov 25;16:1690231. doi: 10.3389/fneur.2025.1690231 (PMC12685712; doi:10.3389/fneur.2025.1690231)
Supplement: Supplementary file 1 [file Data_Sheet_1.DOCX]

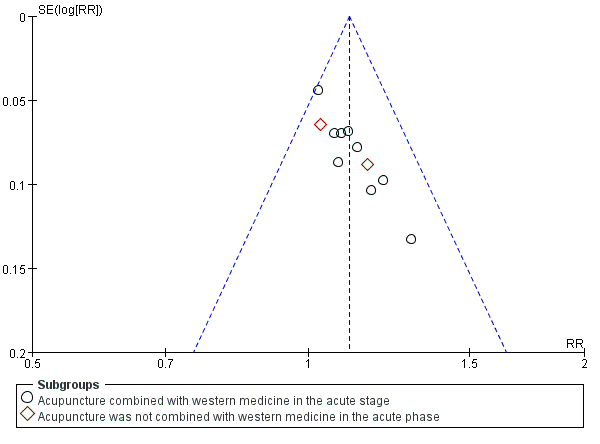


Figure S1. Funnel plot for clinical effective rate


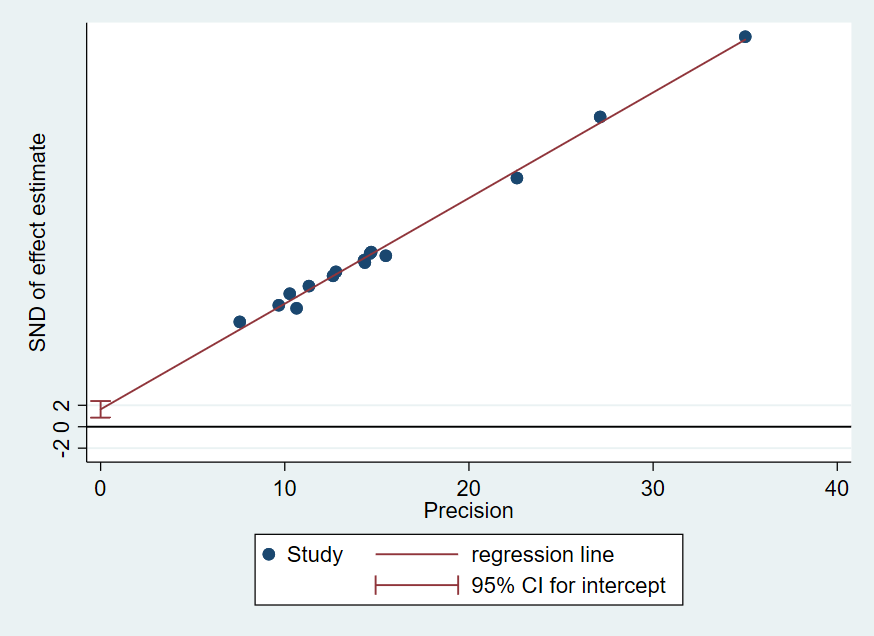


Figure S2. Egger’s test graph for clinical effective rate


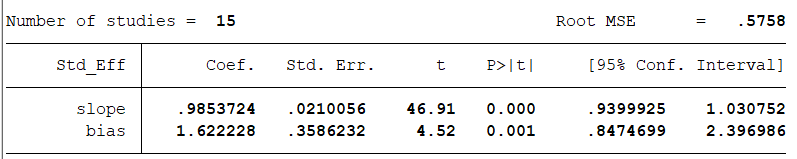


Figure S3. Egger’s test for clinical effective rate


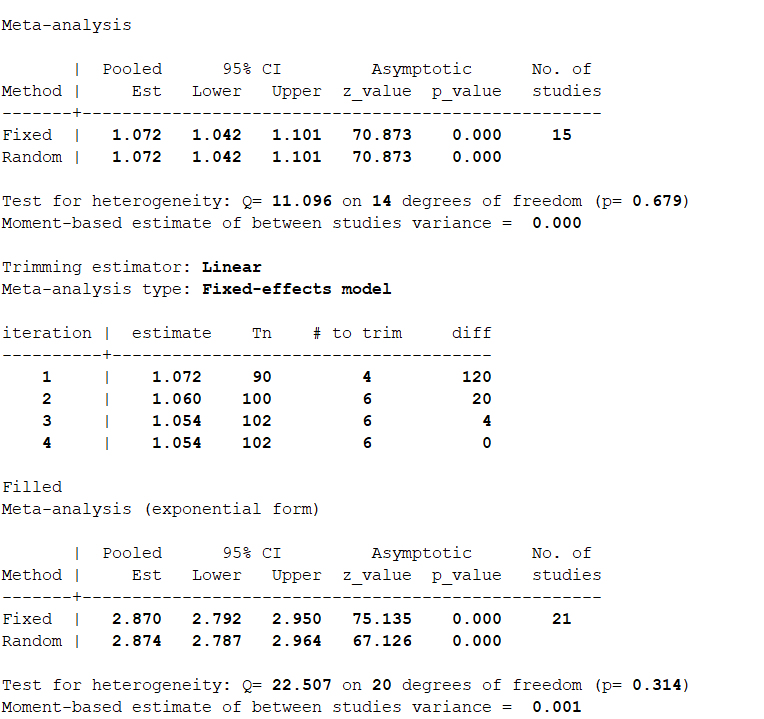


Figure S4. The trim-and-fill method for clinical effective rate


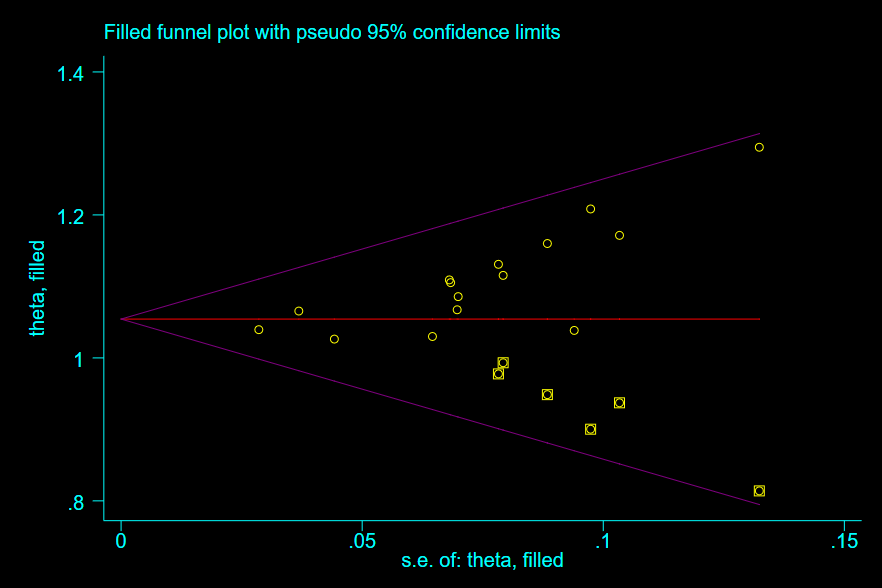


Figure S5. The trim-and-fill method graph for clinical effective rate


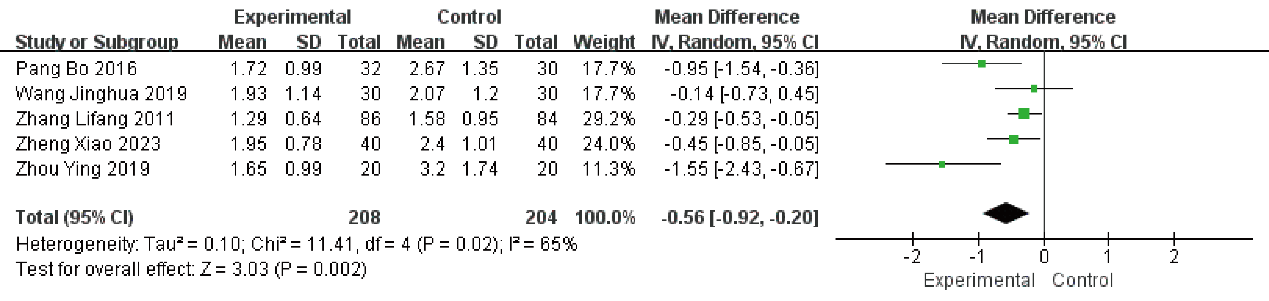


Figure S6. Forest plot for H-B scale scores before subgroup analysis


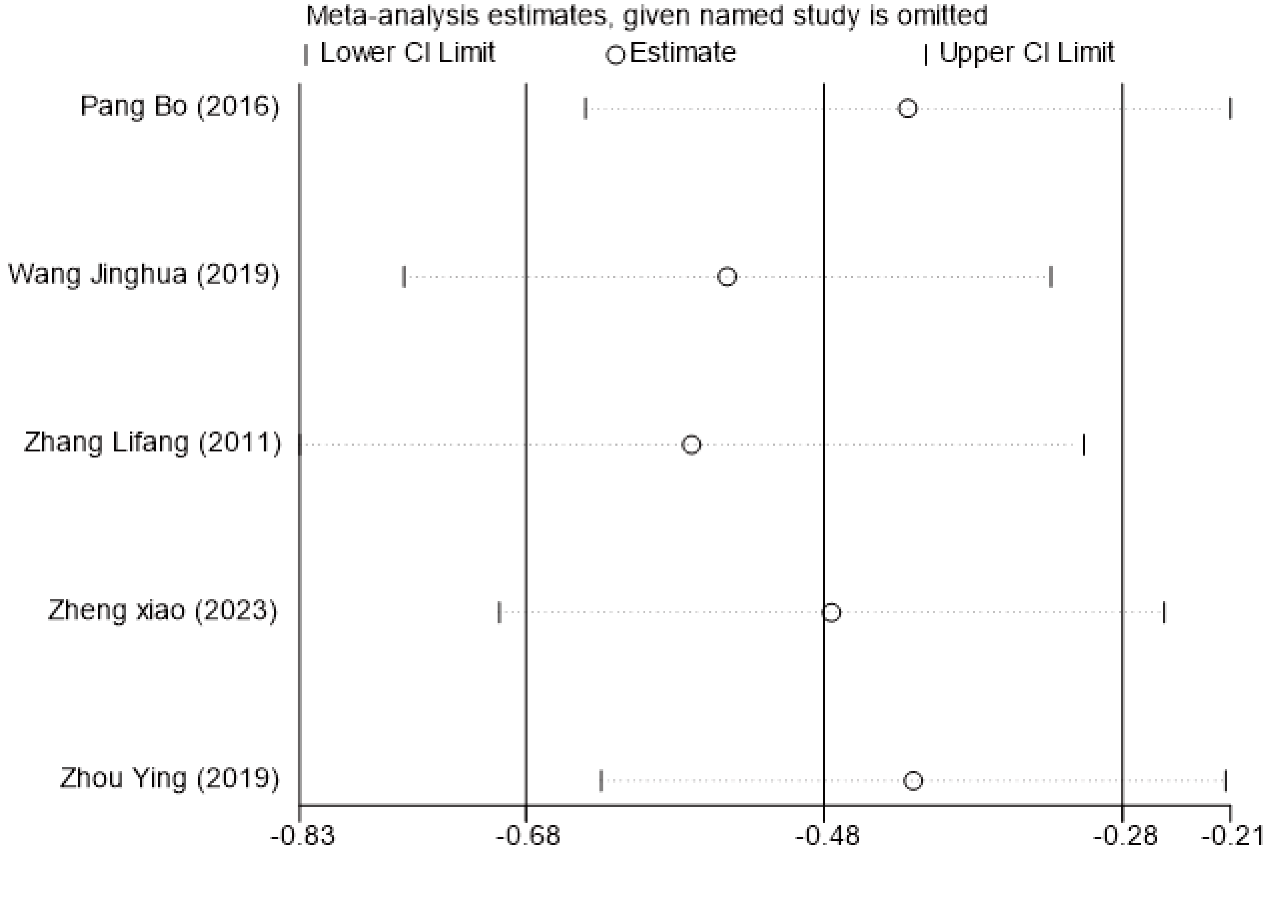


Figure S7. Sensitivity analysis for H-B scale scores before subgroup analysis


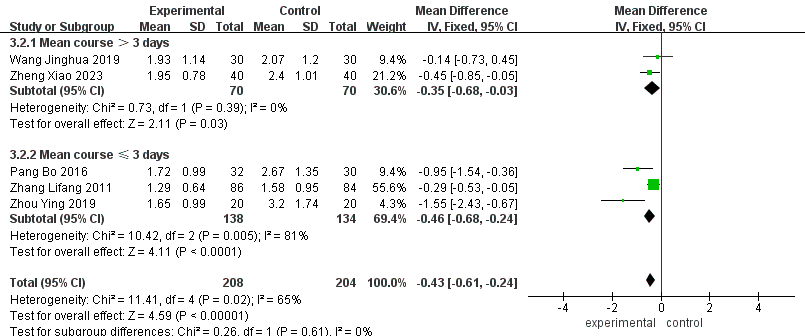


Figure S8. Forest plot for H-B scale scores after subgroup analysis


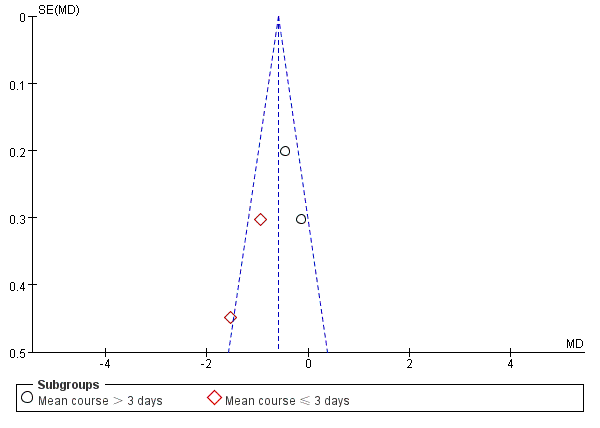


Figure S9. Funnel plot for H-B scale scores


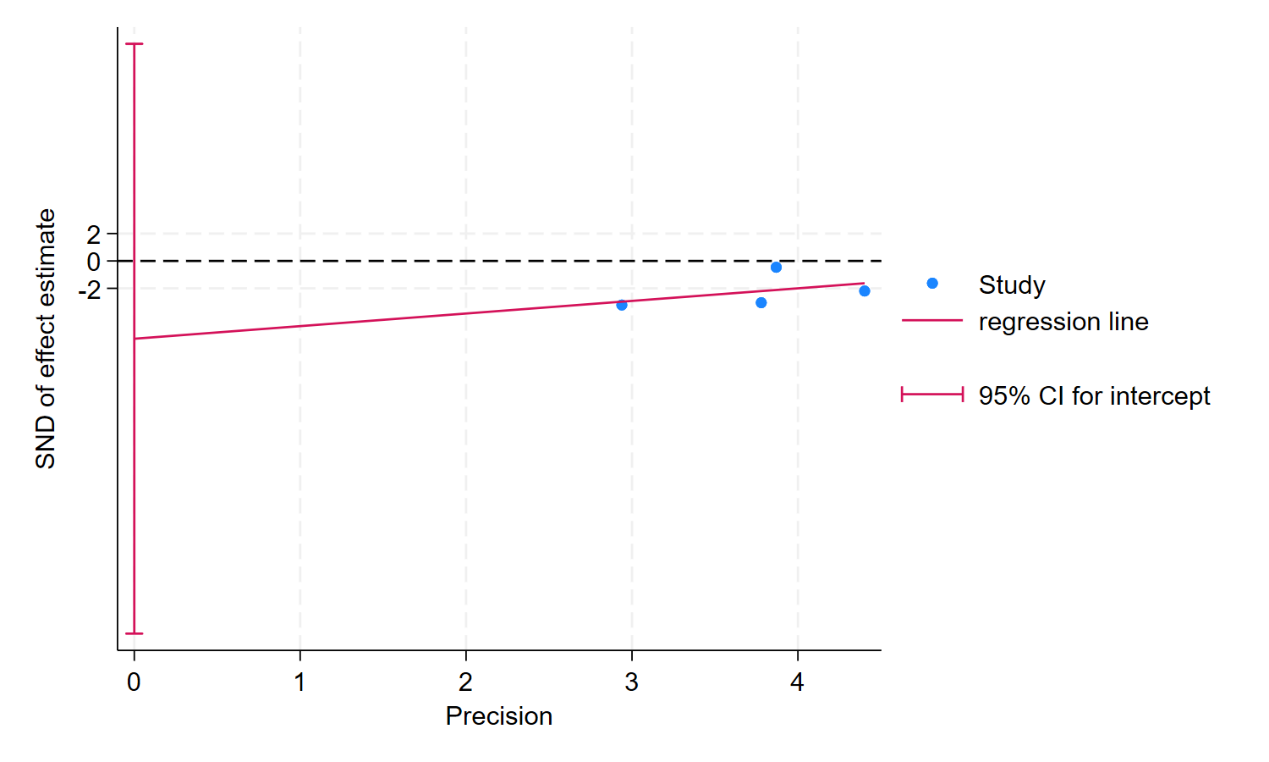


Figure S10. Egger’s test graph for H-B scale scores


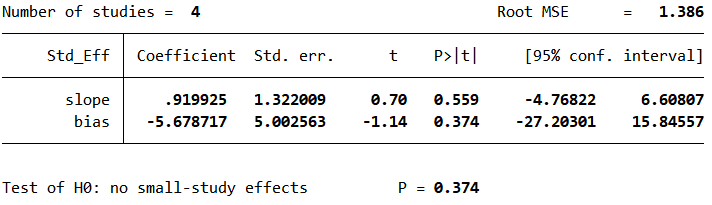


Figure S11. Egger’s test for H-B scale scores


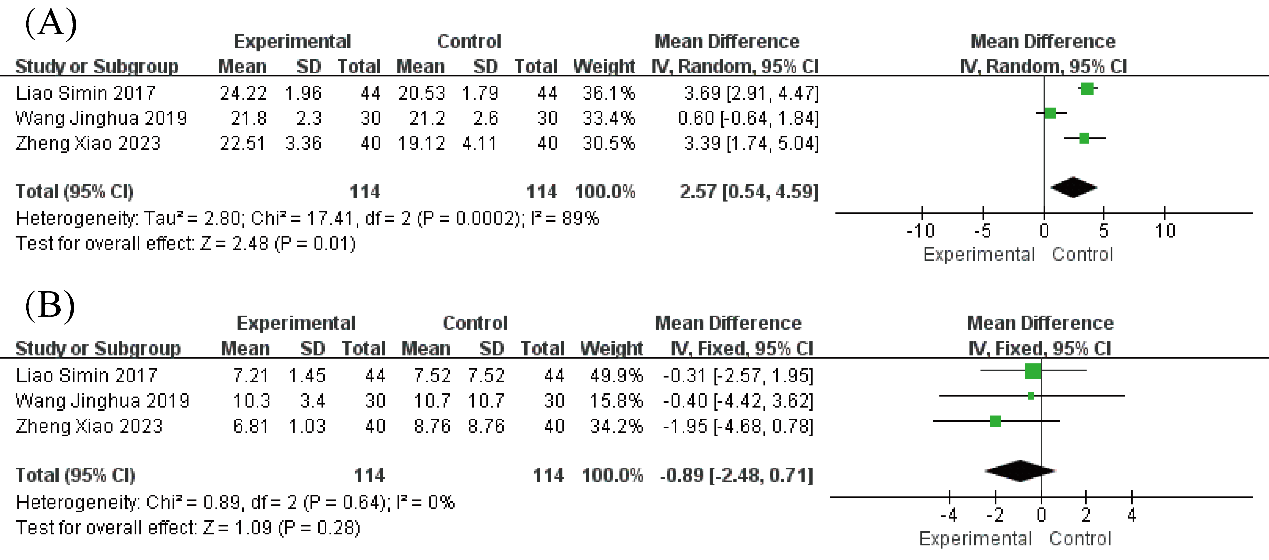


Figure S12. Forest plot for FDI scores before sensitivity analysis


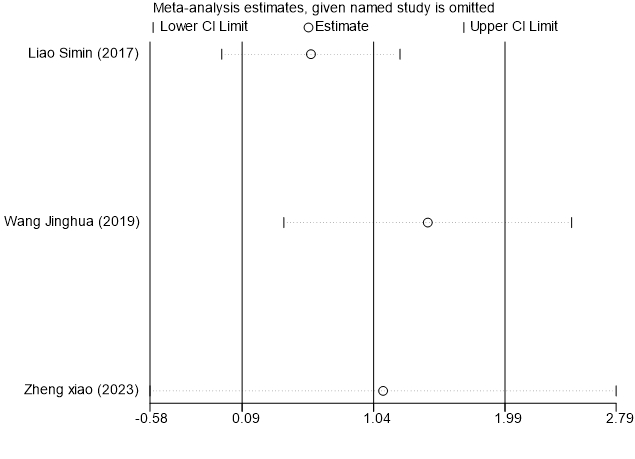


Figure S13. Sensitivity analysis for FDIP scores before sensitivity analysis


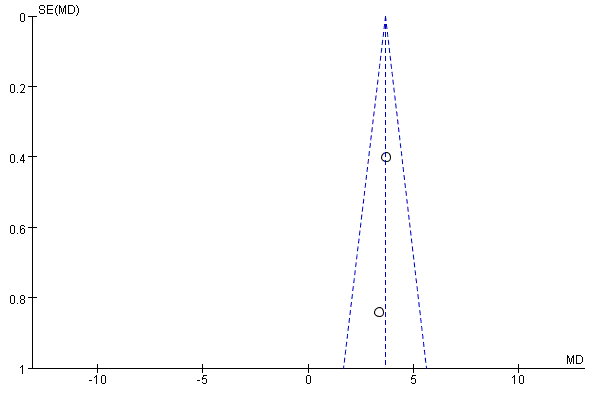


Figure S14. Funnel plot for FDIP scores


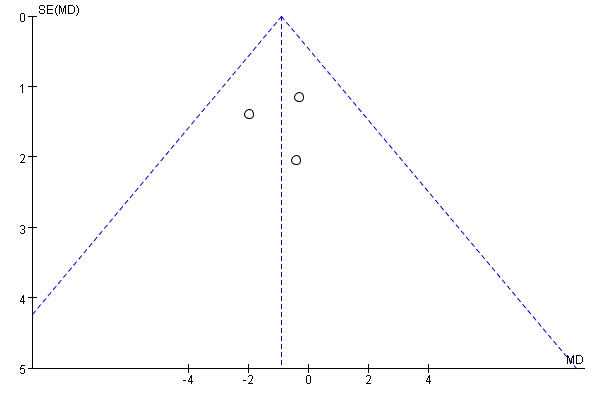


Figure S15. Funnel plot for FDIS scores


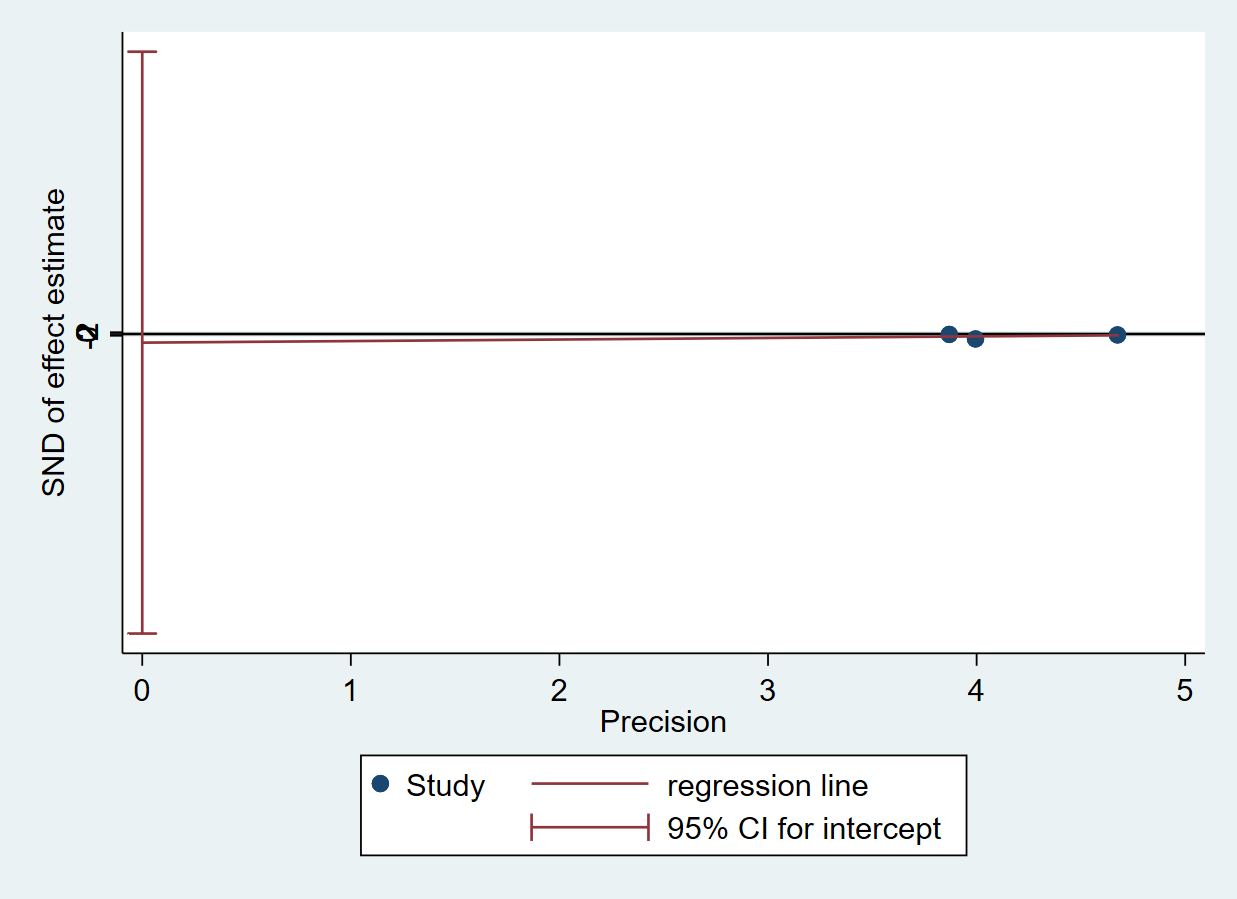


Figure S16. Egger’s test graph for FDIS scores


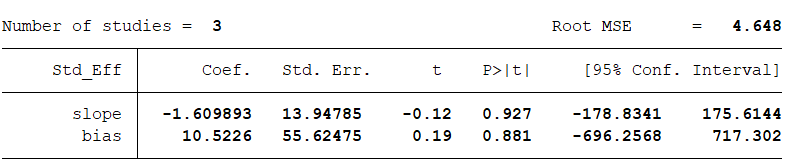


Figure S17. Egger’s test for FDIS scores


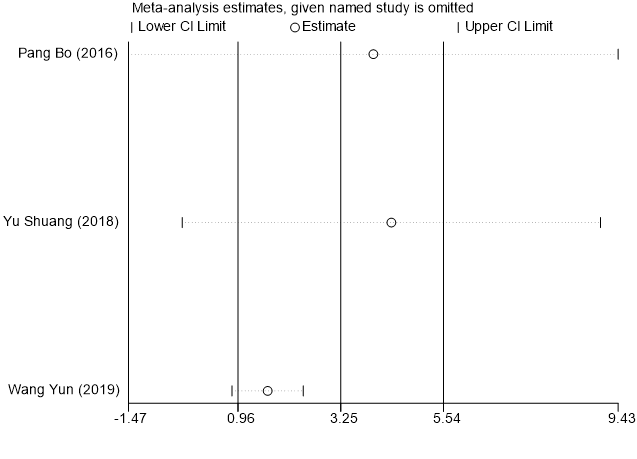


Figure S18. Sensitivity analysis for Portmann score on 28 days after onset


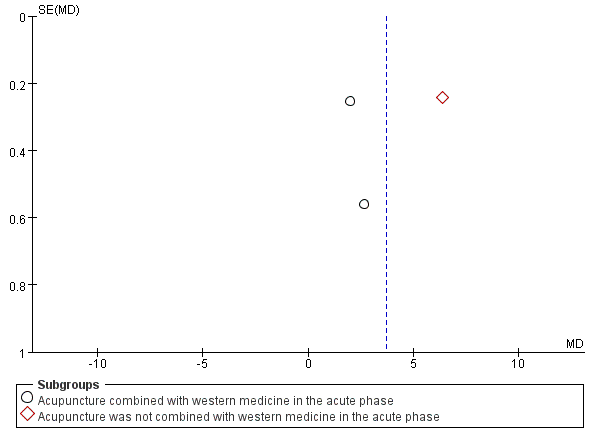


Figure S19. Funnel plot for Portmann Score on 28 days after onset


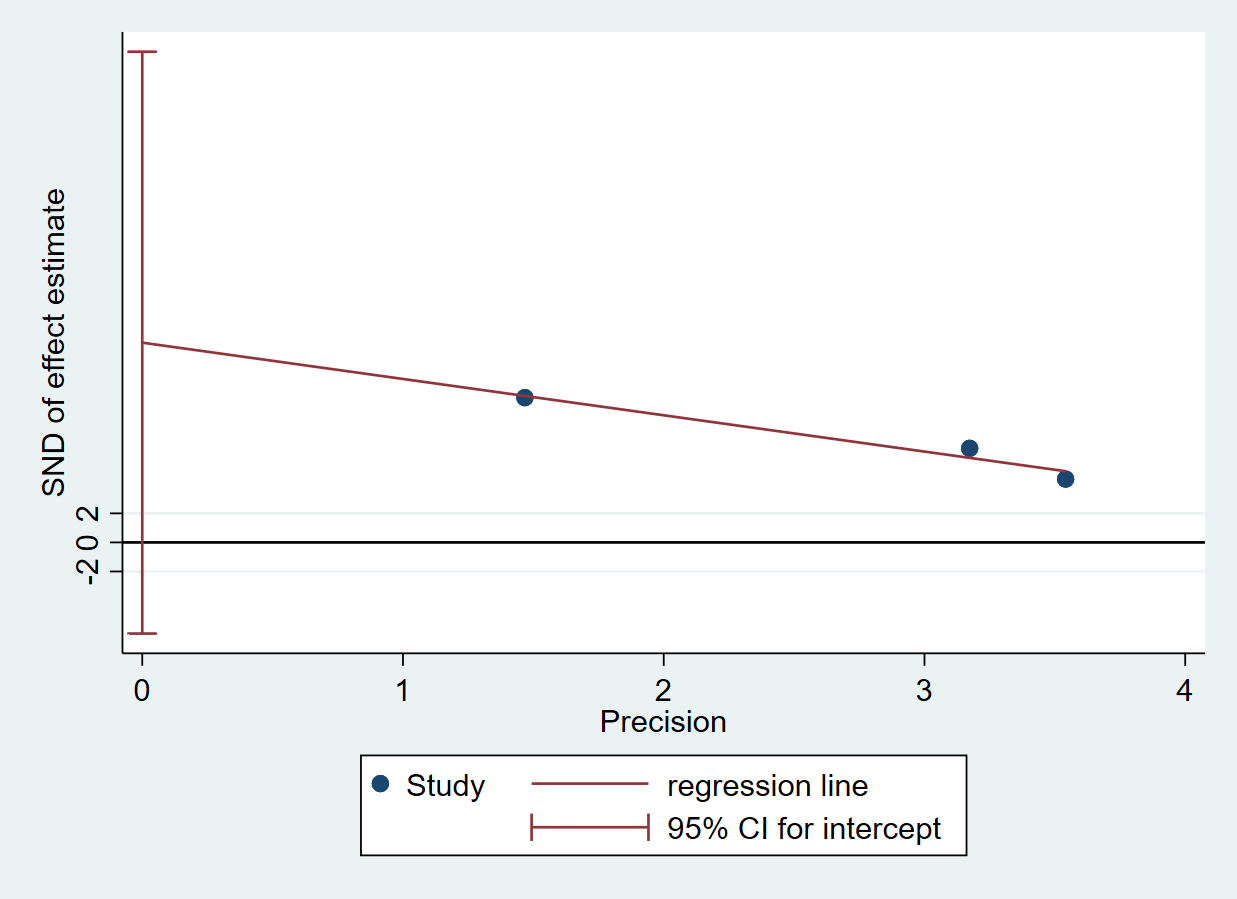


Figure S20. Egger’s test graph for Portmann Score on 28 days after onset


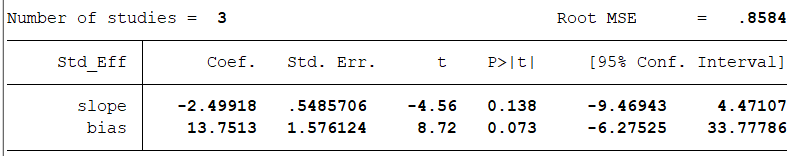


Figure S21. Egger’s test for Portmann Score on 28 days after onset


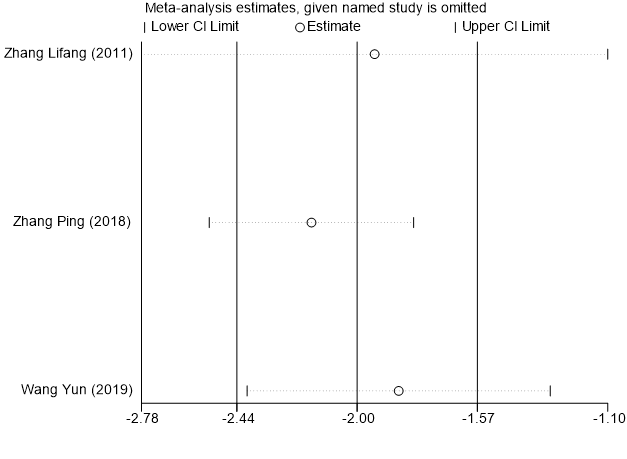


Figure S22. Sensitivity analysis for cure time


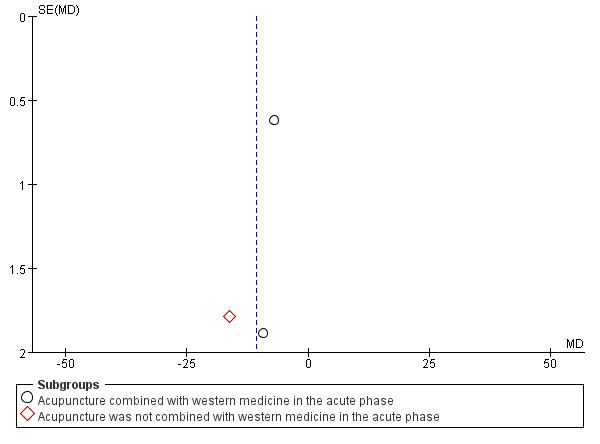


Figure S23. Funnel plot for cure time


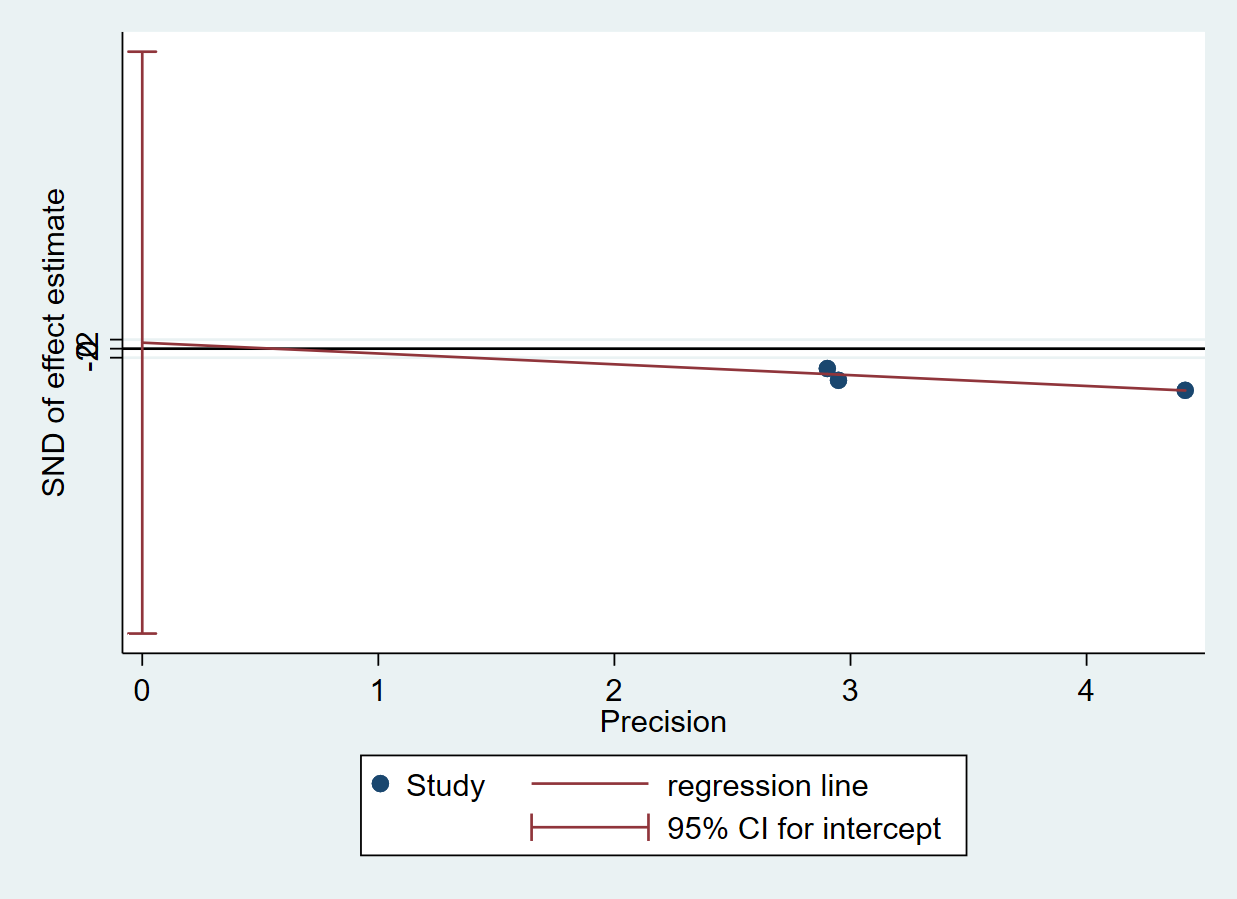


Figure S24. Egger’s test graph for cure time


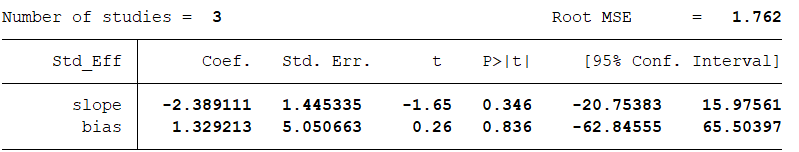


Figure S25. Egger’s test for cure time
