# Supplementary material for: Comparison of acupuncture intervention from the acute phase or the non-acute phase in patients with peripheral facial paralysis: a systematic review and meta-analysis
Source: Front Neurol. 2025 Nov 25;16:1690231. doi: 10.3389/fneur.2025.1690231 (PMC12685712; doi:10.3389/fneur.2025.1690231)
Supplement: Supplementary file 2 [file Table_1.DOCX]

Table S1. Search Strategies

| **Pubmed** | |
| --- | --- |
| #1 | ("Acupuncture"[Mesh]) OR ("Acupuncture Therapy"[Mesh]) OR ("Acupuncture Points"[Mesh]) OR (Acupuncture[Title/Abstract]) OR (Acupoint*[Title/Abstract]) OR (needle[Title/Abstract]) |
| #2 | ("Facial Paralysis"[Mesh]) OR ("Bell Palsy"[Mesh]) OR (facial nerve paralysis[Title/Abstract]) OR (peripheral facial paralysis[Title/Abstract]) OR (bell palsy[Title/Abstract]) OR (facial neuritis[Title/Abstract]) OR (idiopathic facial paralysis[Title/Abstract]) |
| #3 | ("Randomized Controlled Trial"[Publication Type]) OR ("Clinical Trial"[Publication Type]) OR (Trial*[Title/Abstract]) OR (Randomly[Title/Abstract]) OR (Randomized[Title/Abstract]) |
| #4 | #1 AND #2 AND #3 |
| 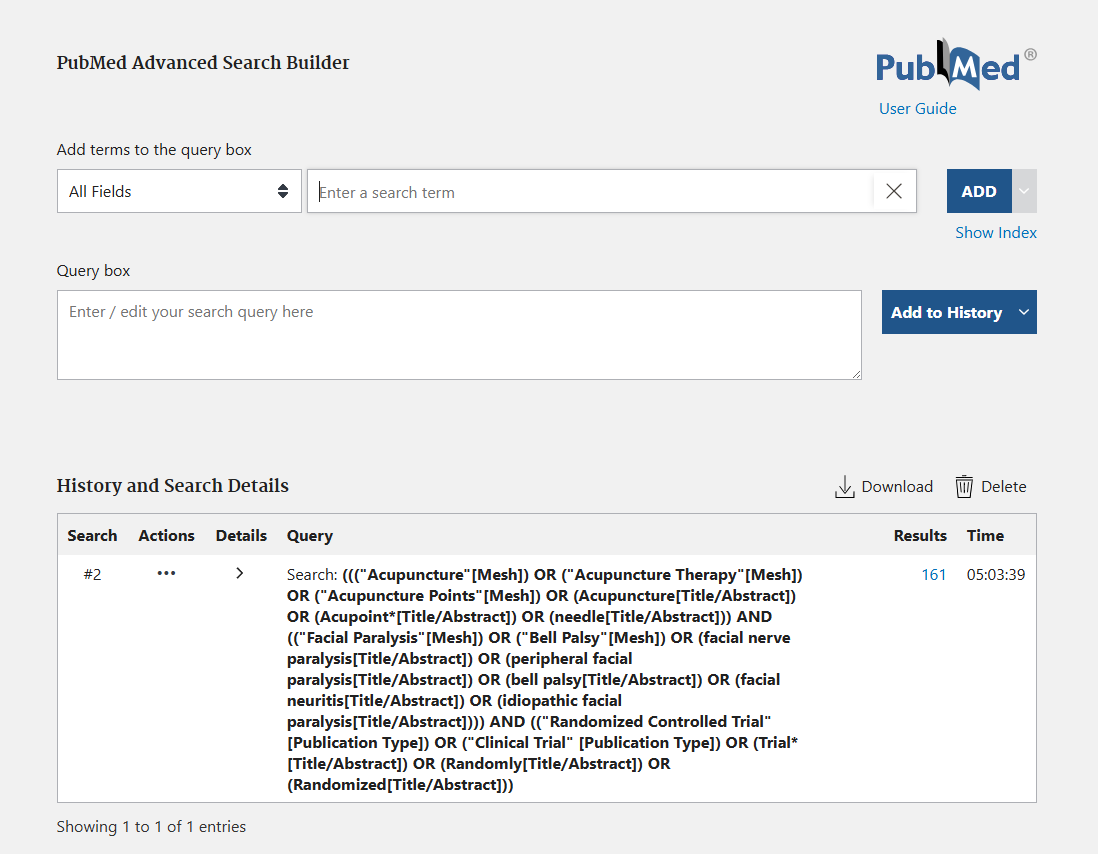 | |
| **Embase** | |
| #1 | 'acupuncture'/exp OR 'acupuncture therapy'/exp OR 'acupuncture':ab,kw,ti OR 'Acupoint*':ab,kw,ti OR 'needle':ab,kw,ti |
| #2 | 'facial nerve paralysis'/exp OR 'facial nerve paralysis':ab,kw,ti OR 'peripheral facial paralysis':ab,kw,ti OR 'bell palsy':ab,kw,ti OR 'facial neuritis':ab,kw,ti OR 'idiopathic facial paralysis':ab,kw,ti |
| #3 | 'randomized controlled trial'/exp OR 'Trial*':ab,kw,ti OR 'Randomly':ab,kw,ti OR 'Randomized':ab,kw,ti |
| #4 | #1 AND #2 AND #3 |
| 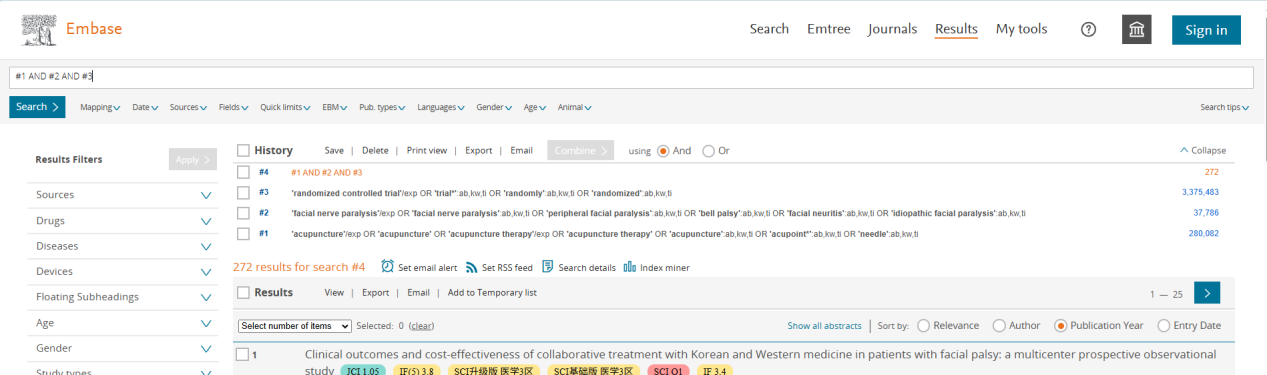 | |
| **Web of Science** | |
| #1 | TS=(acupuncture OR Acupoint* OR needle) |
| #2 | TS=(facial nerve paralysis OR peripheral facial paralysis OR bell palsy OR facial neuritis OR idiopathic facial paralysis) |
| #3 | TS=(randomized controlled trial OR trial* OR Randomly OR Randomized) |
| #4 | #1 AND #2 AND #3 |
| 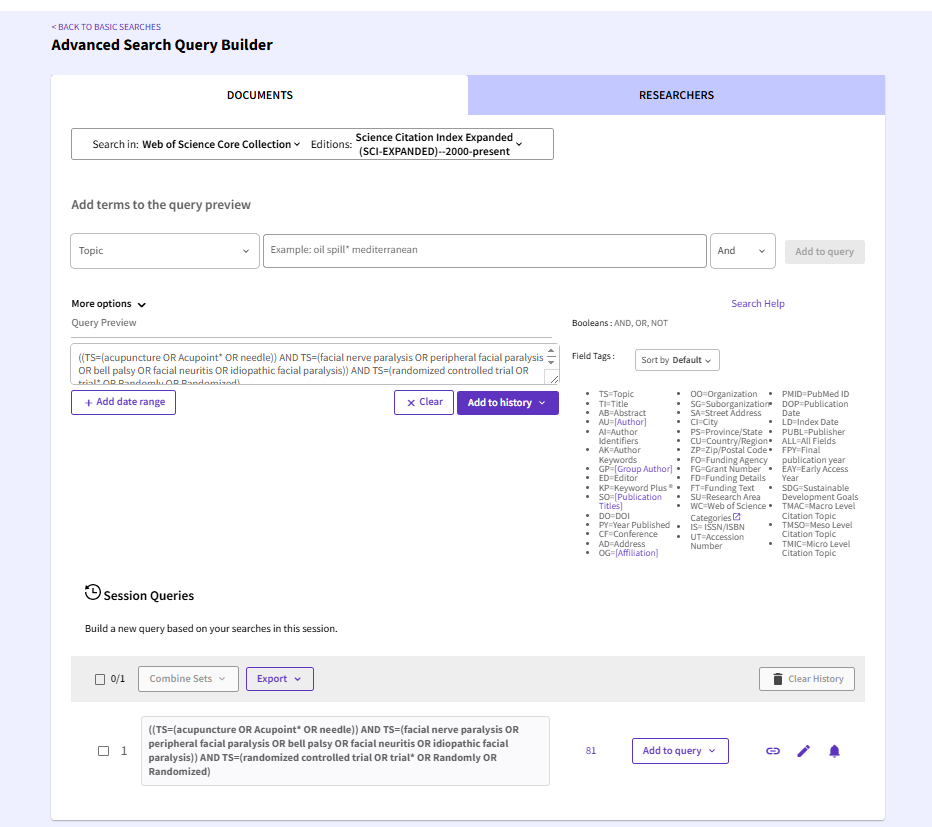 | |
| **Cochrance** | |
| #1 | MeSH descriptor: [Acupuncture Therapy] explode all trees |
| #2 | Acupuncture OR Acupoint* OR needle |
| #3 | #1 OR #2 |
| #4 | MeSH descriptor: [Facial Paralysis] explode all trees |
| #5 | MeSH descriptor: [Bell Palsy] explode all trees |
| #6 | peripheral facial paralysis OR bell palsy OR facial neuritis OR idiopathic facial paralysis OR facial nerve paralysis |
| #7 | #4 OR #5 OR #6 |
| #8 | MeSH descriptor: [Randomized Controlled Trial] explode all trees |
| #9 | randomized controlled trial OR clinical trial OR trial OR randomly OR randomized |
| #10 | #8 OR #9 |
| #11 | #3 AND #7 AND #10 |
| 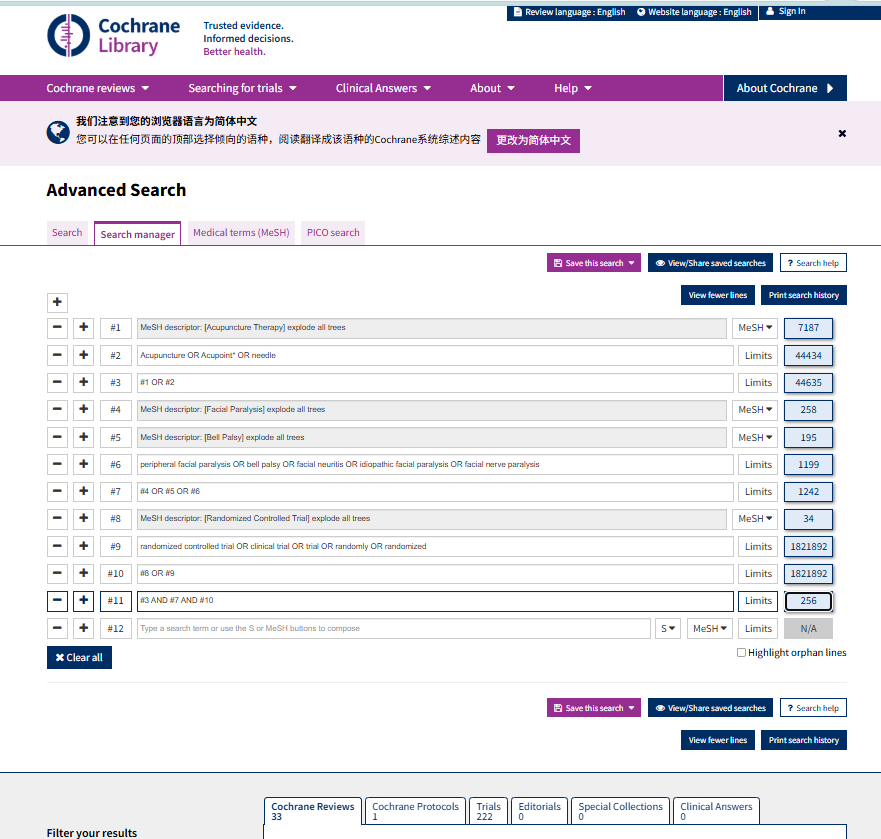 | |
| **China National Knowledge Infrastructure** | |
| #1 | TKA = ‘针灸’ + ‘针刺’ + ‘手针’ + ‘毫针’ + ‘体针’ + ‘针法’ + ‘刺法’ |
| #2 | TKA = (‘周围性面瘫’ + ‘周围性面神经麻痹’ + ‘特发性面神经麻痹’ + ‘贝尔麻痹’ + ‘贝尔氏面瘫’ + ‘面神经麻痹’ + ‘面神经炎’) * (‘急性期’ + ‘急性’) |
| #3 | TKA = ‘随机对照试验’ + ‘试验’ + ‘临床’ + ‘RCT’ + ‘疗效’ + ‘随机’ + ‘对照’ |
| #4 | #1 AND #2 AND #3 |
| 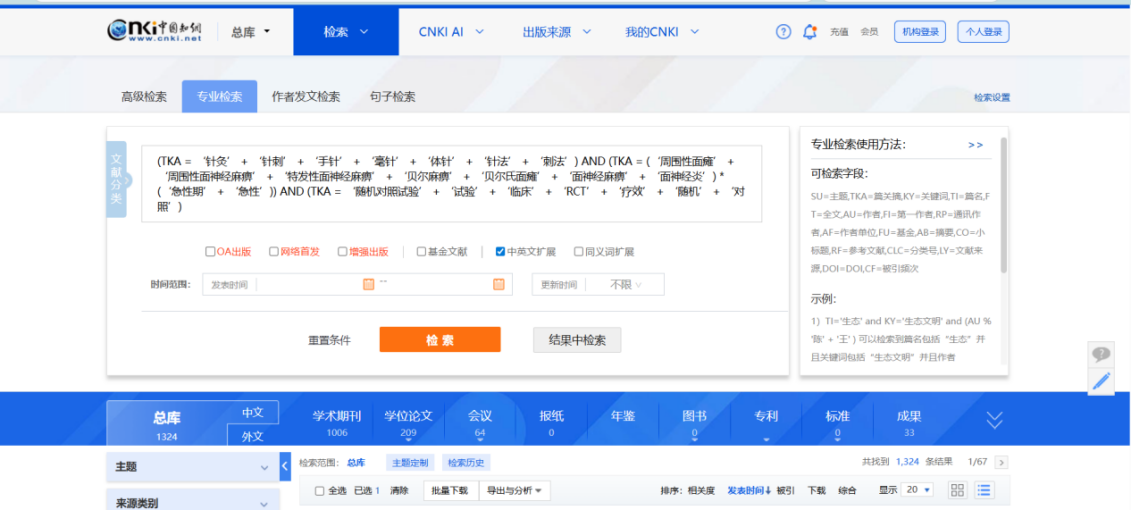 | |
| **China BioMedical Literature Database** | |
| #1 | 摘要 = (“针灸” OR “针刺” OR “手针” OR “毫针” OR “体针” OR “针法” OR “刺法”) |
| #2 | 摘要 = (“周围性面瘫” OR “周围性面神经麻痹” OR “特发性面神经麻痹” OR “贝尔麻痹” OR “贝尔氏面瘫” OR “面神经麻痹” OR “面神经炎”) |
| #3 | 摘要 = (“急性” OR “急性期”) |
| #4 | 摘要 = (“随机对照试验” OR “试验” OR “临床” OR “RCT” OR “疗效” OR “随机” OR “对照”) |
| #5 | #1 AND #2 AND #3 AND #4 |
| 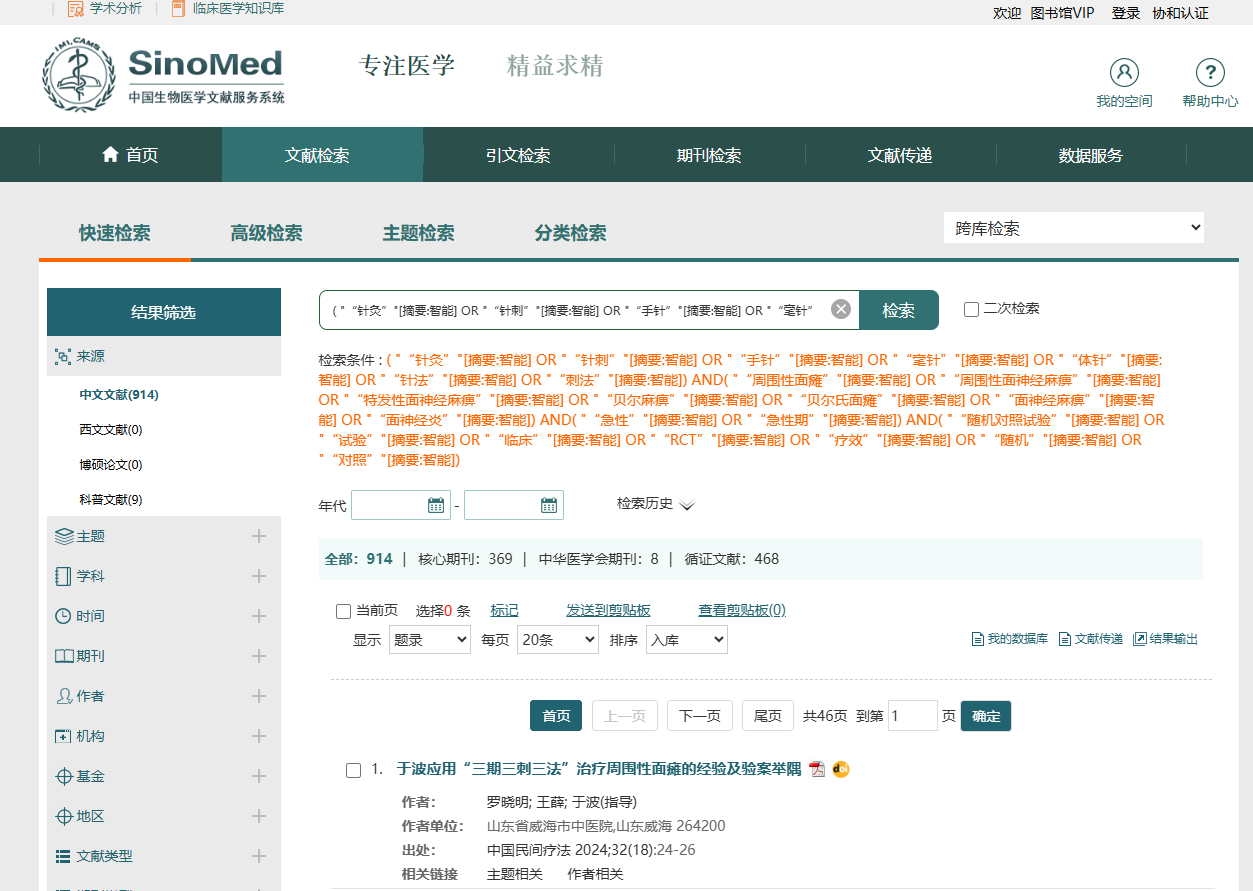 | |
| **Wanfang Digital Journals** | |
| #1 | 摘要= (“针灸” OR “针刺” OR “手针” OR “毫针” OR “体针” OR “针法” OR “刺法”) |
| #2 | 摘要= (“周围性面瘫” OR “周围性面神经麻痹” OR “特发性面神经麻痹” OR “贝尔麻痹” OR “贝尔氏面瘫” OR “面神经麻痹” OR “面神经炎”) AND (“急性” OR “急性期”) |
| #3 | 摘要= (“随机对照试验” OR “试验” OR “临床” OR “RCT” OR “疗效” OR “随机” OR “对照”) |
| #4 | #1 AND #2 AND #3 |
| 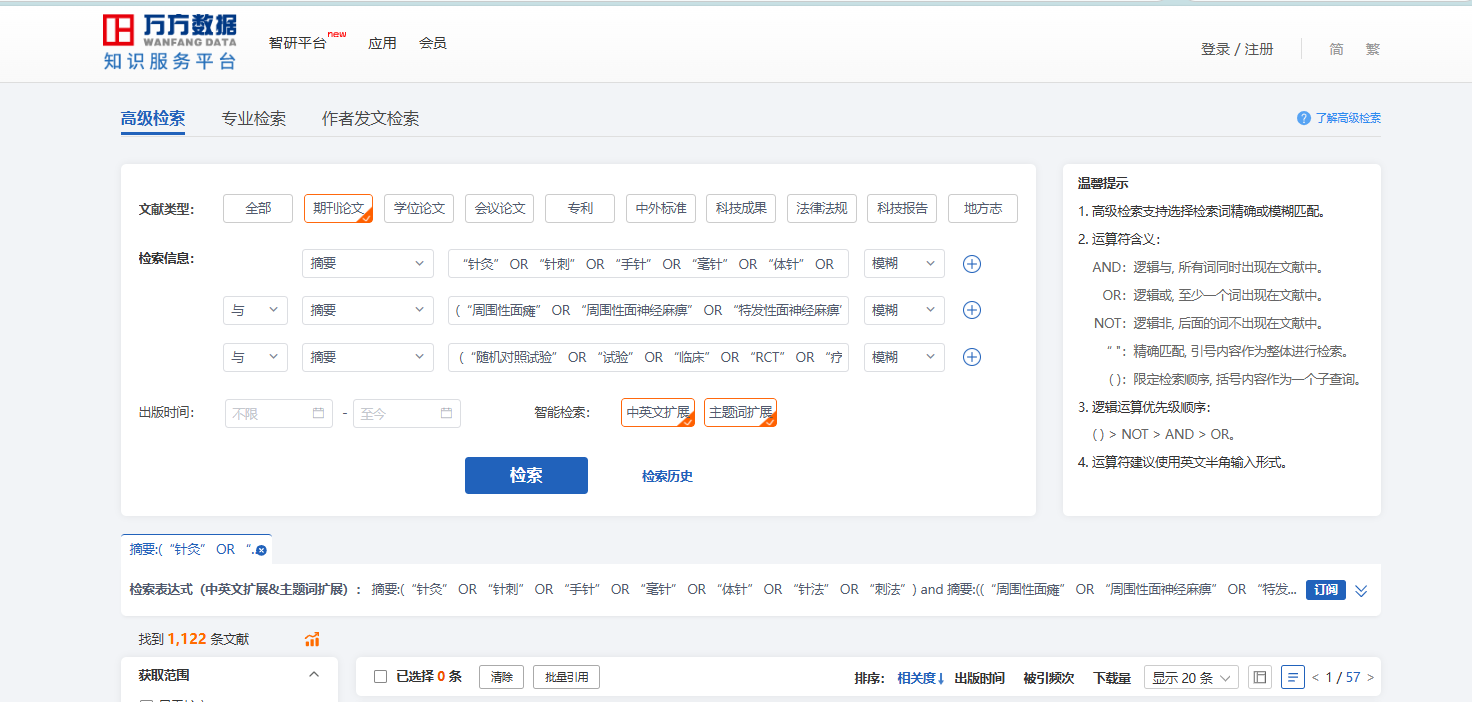 | |
| **VIP database for Chinese Technical Periodicals** | |
| #1 | 摘要 = (针灸 OR 针刺 OR 手针 OR 毫针 OR 体针 OR 针法 OR 刺法) |
| #2 | 摘要 = (周围性面瘫 OR 周围性面神经麻痹 OR 特发性面神经麻痹 OR 贝尔麻痹 OR 贝尔氏面瘫 OR 面神经麻痹 OR 面神经炎) |
| #3 | 摘要 = (急性 OR 急性期) |
| #4 | 摘要 = (随机对照试验 OR 试验 OR 临床 OR RCT OR 疗效 OR 随机 OR 对照) |
| #5 | #1 AND #2 AND #3 AND #4 |
| 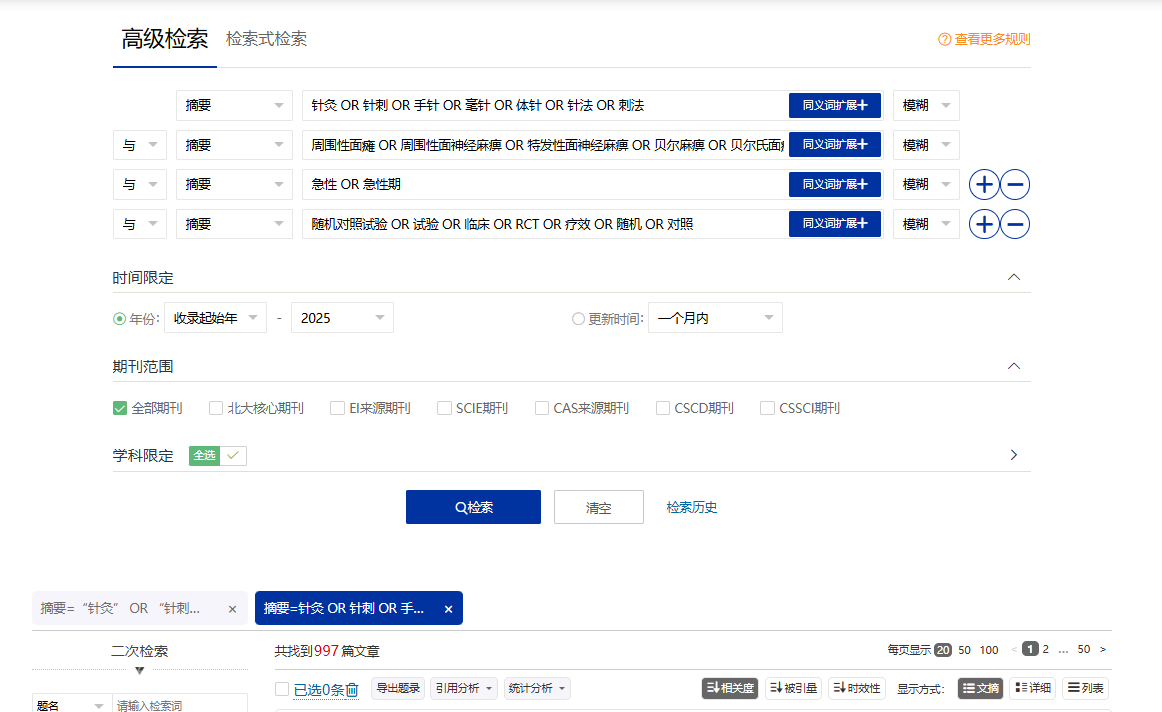 | |

Table S2 GRADE evidence profile for the studies in the meta-analysis

| **Certainty assessment** | | | | | | | **№ of patients** | | **Effect** | | **Certainty** | **Importance** |
| --- | --- | --- | --- | --- | --- | --- | --- | --- | --- | --- | --- | --- |
| **№ of studies** | **Study design** | **Risk of bias** | **Inconsistency** | **Indirectness** | **Imprecision** | **Other considerations** | **acupuncture intervention from the acute phase** | **acupuncture intervention from the non-acute phase** | **Relative (95% CI)** | **Absolute (95% CI)** |  |  |
| **Clinical effective rate** | | | | | | | | | | | | |
| 15 | randomised trials | serious^a^ | not serious | not serious | not serious | none^b^ | 372/391 (95.1%) | 326/380 (85.8%) | **RR 1.11** (1.06 to 1.16) | **94 more per 1,000** (from 51 more to 137 more) | ⨁⨁⨁◯ Moderate^a,b^ | CRITICAL |
| **Non-cure rate at 1-month follow-up** | | | | | | | | | | | | |
| 2 | randomised trials | serious^a^ | serious^c^ | not serious | serious^d^ | none | 16/83 (19.3%) | 22/78 (28.2%) | **RR 0.63** (0.21 to 1.91) | **104 fewer per 1,000** (from 223 fewer to 257 more) | ⨁◯◯◯ Very low^a,c,d^ | IMPORTANT |
| **House-Brackmann Facial Nerve Grading Scale** | | | | | | | | | | | | |
| 5 | randomised trials | not serious | serious^c^ | not serious | not serious | none | 208 | 204 | - | MD **0.56 lower** (0.92 lower to 0.2 lower) | ⨁⨁⨁◯ Moderate^c^ | IMPORTANT |
| **Facial Disability Index-Physical** | | | | | | | | | | | | |
| 3 | randomised trials | not serious | serious^c^ | not serious | serious^d^ | none | 114 | 114 | - | MD **2.57 higher** (0.54 higher to 4.59 higher) | ⨁⨁◯◯ Low^c,d^ | IMPORTANT |
| **Facial Disability Index-Social** | | | | | | | | | | | | |
| 3 | randomised trials | not serious | not serious | not serious | serious^d^ | none | 114 | 114 | - | MD **0.89 lower** (2.48 lower to 0.71 higher) | ⨁⨁⨁◯ Moderate^d^ | IMPORTANT |
| **Portmann Simple Score Scale on 7 days after onset** | | | | | | | | | | | | |
| 2 | randomised trials | not serious | serious^c^ | not serious | serious^d^ | none | 62 | 60 | - | MD **3.05 higher** (0.55 higher to 5.55 higher) | ⨁⨁◯◯ Low^c,d^ | IMPORTANT |
| **Portmann Simple Score Scale on 14 days after onset** | | | | | | | | | | | | |
| 2 | randomised trials | not serious | serious^c^ | not serious | serious^d^ | none | 62 | 60 | - | MD **3.42 higher** (1.78 higher to 5.07 higher) | ⨁⨁◯◯ Low^c,d^ | IMPORTANT |
| **Portmann Simple Score Scale on 28 days after onset** | | | | | | | | | | | | |
| 3 | randomised trials | not serious | serious^c^ | not serious | serious^d^ | none | 92 | 90 | - | MD **3.69 higher** (0.5 higher to 6.87 higher) | ⨁⨁◯◯ Low^c,d^ | IMPORTANT |
| **Cure time** | | | | | | | | | | | | |
| 3 | randomised trials | not serious | serious^c^ | not serious | serious^d^ | none | 122 | 104 | - | MD **10.71 lower** (16.33 lower to 5.09 lower) | ⨁⨁◯◯ Low^c,d^ | IMPORTANT |
| **CI**: confidence interval; **MD**: mean difference; **RR**: risk ratio  **Explanations:**  a. The included studies have an unclear risk of bias.  b. The Egger test results indicate that there is a significant publication bias in this evidence, but the results of the trim-and-fill method show that the combined effect size is robust, indicating that the evidence is not significantly affected by publication bias.  c. high heterogeneity  d. The sample size was relatively small | | | | | | | | | | | | |
